# Solutions of fuzzy advection-diffusion and heat equations by natural adomian decomposition method

**DOI:** 10.1038/s41598-023-45207-y

**Published:** 2023-10-30

**Authors:** Noor Jamal, Muhammad Sarwar, Parveen Agarwal, Nabil Mlaiki, Ahmad Aloqaily

**Affiliations:** 1 Department of Mathematics, University of Malakand, Dir Lower, Pakistan; 2 Nonlinear Dynamics Research Center (NDRC), Ajman University, Ajman, UAE; 3 Department of Mathematics, Anand International College of Engineering Near Kanota, Agra Road, Jaipur, Rajasthan 303012 India; 4 Department of Mathematics and General Sciences, Price Sultan University, P.O. Box 66833, Riyadh, 11586 Saudi Arabia

**Keywords:** Engineering, Mathematics and computing

## Abstract

In this article, we present an algorithm for computing analytical solutions of linear fuzzy advection-diffusion equations and one-dimensional fuzzy heat equations involving an external source. The fuzzy problems can be solved by using the natural transform and Adomian decomposition method. The results obtained through the natural Adomian decomposition method are calculated in a series form that converges rapidly to the exact solution. To enhance the practicality of our work, we provide examples to illustrate our findings.

## Introduction

Zadeh^1^ introduced the concept of fuzzy sets. Chang and Zadeh utilized fuzzy sets to introduce fuzzy mappings and control^2^. The foundation of elementary fuzzy calculus was laid by the work of several researchers on fuzzy mappings^3–6^. In the recent years, there has been growing interest among researchers in applying these concepts to fuzzy differential and integral equations in the field of physical sciences. While studying natural phenomena, partial differential equations (PDEs) are more relevant as  compared to ordinary differential equations because they deal with multiple variables simultaneously. The significant role of PDEs can be observed in the important fields like physics, biology, engineering etc. However, in certain cases, the fuzziness of real-life problems, such as the initial values of partial differential dynamical systems makes PDEs less suitable. Buckley and Feuring^7^ addressed this limitation by incorporating fuzzy set theory into partial differential equations, by introducing fuzzy partial differential equations. Allahviranloo^8^ solved fuzzy partial differential equations(FPDEs) using difference methods based on the Taylor series. Pownuk^9^ obtained solutions for FPDEs through sensitivity analysis and the finite element method. Various methods, such as integral transforms (Fourier, Laplace, Sumudu, etc.) iterative procedures, series solution methods (Homotopy, Adomian decomposition, Laplace Adomian decomposition methods, etc.) and Taylor’s series method have been employed to address fuzzy problems. However, to the best of our knowledge these methods have not been extensively utilized to tackle FPDEs. Integral transform methods pose challenges when the fuzzy function is (i)-differentiable with respect to one variable and (ii)-differentiable with respect to the second variable. Adomian decomposition method(ADM) was introduced by George Adomian^10^. To solve nonlinear ordinary differential equations and PDEs. The solution of ADM is in the form of infinite series which rapidly converges to an accurate solution. For the solutions of differential equations, a lot of mathematicians used ADM and various modifications of ADM see Refs.^11–19^. The natural transforms, was first introduced by Khan and Khan^20^, which have the unique ability to converge to both Laplace and Sumudu transforms by simply changing the variables. Therefore natural transform is used as a checker on the Laplace and Sumudu transforms. For basic concept of natural transform method intrusted readers can see Refs.^21–24^. The advection equations and advection-diffusion equations have been solved by the authors of Refs.^25–28^ with different methods. While, heat equations has been extensively investigated by various authors using different dimensions and techniques, as detailed in Refs.^29–36^.

In the current study, we use the natural Adomian decomposition method to compute the solutions of fuzzy partial differential equations(FPDEs). By addressing the complexities associated with integral transforms used for the solutions of FPDEs, specifically, we apply the natural Adomian decomposition method to solve fuzzy advection-diffusion equations and heat equations involving an external source.

## Preliminaries

In the sequel, we need the following definitions and results from the existing literature.

Let $R_F$ be the class of fuzzy subsets. The membership functions, $k:R \rightarrow [0,1],$ satisfying the following conditions

(i) *k* is upper semi continuous;
(ii) *k* $$\{{\mu y_1 + (1-\mu )y_2}\}\ge min\{{k(y_1),k(y_2)\}}, y_1, y_2 \in R\ \hbox {and}\ \mu \in [0,1]$$;
(iii) *k* is normal, so $\exists$ $y_{0}$ $\in R$ such that $k (y_0)=1$;
(iv) $cl\{y \ \in R$, $k(y)>0\}$ is compact where cl denotes the closure of a subset.

Then $R_F$ is called the space of fuzzy numbers.

Let $[v]^\alpha = \{a\in R \ | v(a)\ge \alpha \}$ is $\alpha -$level set where $[v]^\alpha \in R_F,$ for all $0\le \alpha \le 1.$ The following Remark shows that the notation of $\alpha -$level set $[v]^\alpha = [{\underline{v}}^{\alpha }, {\overline{v}}^{\alpha }]$ is a valid $\alpha -$level set for some $[v]^\alpha \in R_F.$

### Remark 1.1^37^

 The fuzzy interval $[{\underline{v}}^{\alpha }, {\overline{v}}^{\alpha }]$ is parametric form if the following conditions holds with $0\le \alpha \le 1.$

(i) non-decreasing bounded function, ${\underline{v}}^{\alpha }$ is left-continuous in [0, 1] and right-continuous for $\alpha =0$;
(ii) non-increasing bounded function, ${\overline{v}}^{\alpha }$ is left-continuous in [0, 1] and right-continuous for $\alpha =0$;
(iii) $${\underline{v}}^{\alpha }\le {\overline{v}}^{\alpha }.$$

Let $x, y\in R_{F}$ and $\lambda \in R$ then the usual addition and product are defined with $[x + y]^\alpha = [x]^\alpha + [y]^\alpha$ and $[\lambda x]^\alpha = \lambda [x]^\alpha$ for all $\alpha \in [0,1].$

### Definition 2.1^38^

 The function $d_{F}:R_{F}\times R_{F} \rightarrow R^{+}\cup \{0\},$ define by

$$\begin{aligned} d_{F}(x,y) = \sup _{\alpha \in [0,1]} d(x^\alpha , y^\alpha ) = \sup _{\alpha \in [0,1]} \max \big \{|{\underline{x}}^\alpha - {\underline{y}}^\alpha |,|{\overline{x}}^\alpha - {\overline{y}}^\alpha |\big \}. \end{aligned}$$

It is well known that $(d_{F},R_{F})$ is complete metric space. Moreover, $d_{F}$ satisfy the following properties, $\forall$ $x,\ y,\ z, a \in R_{F}.$

$$D_1 ) \ d_{F}(x+z,y+z) = d_{F}(x,y);$$

$$D_2) \ d_{F}(\alpha x, \alpha y) = \alpha d_{F}(x,y);$$

$$D_3) \ d_{F}(x+a,y+z) \le d_{F}(x,y) + d_{F}(a,z).$$

### Definition 2.2^39^

 Let $x,\ y,\ z \in R_{F}$ such that $x = y + z$ then *z* is $H-$difference of *x*, *y* and denoted by $x\ominus y.$

### Definition 2.3^39, 40^

 Let $\chi :I \rightarrow R_{F},$ where $I = [a,b]$ and $y \in I,$ is generalized differentiable at *y* if there exist, $\chi ^{'}(y)\in R_{F}$ such that either

(i) For sufficiently small $r>0,$ the Hukuhara difference, ${\chi (y+r)\ominus \chi (y)},$ ${\chi (y)\ominus \chi (y-r)}$ and limits exist in $(d_{F},R_{F}).$
  $$\begin{aligned} \lim _ {r \rightarrow 0} \frac{\chi (y)\ominus \chi (y+h)}{r}= & {} \lim _ {r \rightarrow 0} \frac{\chi (y-r)\ominus \chi (y)}{r} = \chi ^{'}(y) \end{aligned}$$
(ii) For sufficiently small $r>0,$ the Hukuhara difference, ${\chi (y)\ominus \chi (y+r)},$ ${\chi (y-r)\ominus \chi (y)}$ and limits exist in $(d_{F},R_{F}).$
  $$\begin{aligned} \lim _ {r \rightarrow 0} \frac{\chi (y+r)\ominus \chi (y)}{(-r)}= & {} \lim _ {r \rightarrow 0} \frac{\chi (y)\ominus \chi (y-r)}{(-r)} = \chi ^{'}(y) \end{aligned}$$
  The first one is referred to $(i)-$differentiable and second one to $(ii)-$differentiable.

### Theorem 2.4^35, 41^

 Let the continuous fuzzy function, $\chi :[a,b] \rightarrow R_{F},$ such that $[\chi (s)]^\alpha = \big [{\underline{\chi }}^\alpha (s), {\overline{\chi }}^\alpha (s)\big ]$, and $0\le \alpha \le 1$.

(i) If $[\chi (s)]^\alpha$ is $(i)-$differentiable then ${\underline{\chi }}^\alpha (s), {\overline{\chi }}^\alpha (s)$ are differentiable and $[\chi ^{'}(s)]^\alpha = \big [({\underline{\chi }}^\alpha )^{'}(s), ({\overline{\chi }}^\alpha )^{'} (s)\big ].$
(ii) If $[\chi (s)]^\alpha$ is $(ii)-$differentiable then ${\underline{\chi }}^\alpha (s), {\overline{\chi }}^\alpha (s)$ are differentiable and $[\chi ^{'}(s)]^\alpha = \big [({\overline{\chi }}^\alpha )^{'}(s), ({{\underline{\chi }}}^\alpha )^{'} (s)\big ].$

### Definition 2.5^39, 40^

 Let $\chi ^{'}:I \rightarrow R_{F},$ where $I = [a,b]$ and $y \in I,$ is generalized differentiable at *y* if there exist, $\chi ^{''}(y)\in R_{F}$ such that either

(i) For sufficiently small $r>0,$ the Hukuhara difference, ${\chi ^{'}(y+r)\ominus \chi ^{'}(y)},$ ${\chi ^{'}(y)\ominus \chi ^{'}(y-r)}$ and limits exist in $(d_{F},R_{F}).$
  $$\begin{aligned} \lim _ {r \rightarrow 0} \frac{\chi ^{'}(y)\ominus \chi ^{'}(y+h)}{r}= & {} \lim _ {r \rightarrow 0} \frac{\chi ^{'}(y-r)\ominus \chi ^{'}(y)}{r} = \chi ^{''}(y) \end{aligned}$$
(ii) For sufficiently small $r>0,$ the Hukuhara difference, ${\chi ^{'}(y)\ominus \chi ^{'}(y+r)},$ ${\chi ^{'}(y-r)\ominus \chi ^{'}(y)}$ and limits exist in $(d_{F},R_{F}).$
  $$\begin{aligned} \lim _ {r \rightarrow 0} \frac{\chi ^{'}(y+r)\ominus \chi ^{'}(y)}{(-r)}= & {} \lim _ {r \rightarrow 0} \frac{\chi ^{'}(y)\ominus \chi ^{'}(y-r)}{(-r)} = \chi ^{''}(y) \end{aligned}$$
  The first one is referred to $(i)-$differentiable and second one to $(ii)-$differentiable.

Now we present partial generalized Hukuhara derivative for fuzzy function^35, 41^

### Definition 2.6

Let $\chi : [a,b]\times [c,d]\rightarrow R_{F},$ is generalized differentiable with respect to *t* at $(t_{0}, z_{0}) \in [a,b]\times [c,d],$ if there exist, $\chi _{t}(t_{0}, z_{0})\in R_{F}$ such that either

(i) For sufficiently small $r>0,$ the Hukuhara difference ${\chi (t_{0}+r, z_{0})\ominus \chi (t_{0}, z_{0})},$ ${\chi (t_{0}, z_{0})\ominus \chi (t_{0}-r, z_{0})}$ and limits exist in $(d_{F},R_{F}).$
  $$\begin{aligned} \lim _ {r \rightarrow 0} \frac{\chi (t_{0}+r, z_{0})\ominus \chi (t_{0}, z_{0})}{r}= & {} \lim _ {r \rightarrow 0} \frac{\chi (t_{0}, z_{0})\ominus \chi (t_{0}-r, z_{0})}{r} = \chi _{t}(t_{0}, z_{0}). \end{aligned}$$
(ii) For sufficiently small $r>0,$ the Hukuhara difference, ${\chi (t_{0}, z_{0})\ominus \chi (t_{0}+r, z_{0})},$ ${\chi (t_{0}-r, z_{0})\ominus \chi (t_{0}, z_{0})}$ ${\chi (y)\ominus \chi (y+r)},$ ${\chi (y-r)\ominus \chi (y)}$ and limits exist in $(d_{F},R_{F}).$
  $$\begin{aligned} \lim _ {r \rightarrow 0} \frac{\chi (t_{0}, z_{0})\ominus \chi (t_{0}+r, z_{0})}{(-r)}= & {} \lim _ {r \rightarrow 0} \frac{\chi (t_{0}-r, z_{0})\ominus \chi (t_{0}, z_{0})}{(-r)} = \chi _{t}(t_{0}, z_{0}). \end{aligned}$$

### Definition 2.7

Let $\chi _{t}: [a,b]\times [c,d]\rightarrow R_{F},$ is generalized differentiable with respect to *t* at $(t_{0}, z_{0}) \in [a,b]\times [c,d],$ if there exist, $\chi _{tt}(t_{0}, z_{0})\in R_{F}$ such that either

(i) For sufficiently small, $r>0,$ the Hukuhara difference ${\chi _{t}(t_{0}+r, z_{0})\ominus \chi _{t}(t_{0}, z_{0})},$ ${\chi _{t}(t_{0}, z_{0})\ominus \chi _{t}(t_{0}-r, z_{0})}$ and limits exist in $(d_{F},R_{F}).$
  $$\begin{aligned} \lim _ {r \rightarrow 0} \frac{\chi _{t}(t_{0}+r, z_{0})\ominus \chi _{t}(t_{0}, z_{0})}{r}= & {} \lim _ {r \rightarrow 0} \frac{\chi _{t}(t_{0}, z_{0})\ominus \chi _{t}(t_{0}-r, z_{0})}{r} = \chi _{tt}(t_{0}, z_{0}). \end{aligned}$$
(ii) For $r>0,$ sufficiently small, the Hukuhara difference ${\chi _{t}(t_{0}, z_{0})\ominus \chi _{t}(t_{0}+r, z_{0})},$ ${\chi _{t}(t_{0}-r, z_{0})\ominus \chi (t_{0}, z_{0})}$ and limits exist in $(d_{F},R_{F}).$
  $$\begin{aligned} \lim _ {r \rightarrow 0} \frac{\chi _{t}(t_{0}, z_{0})\ominus \chi _{t}(t_{0}+r, z_{0})}{(-r)}= & {} \lim _ {r \rightarrow 0} \frac{\chi _{t}(t_{0}-r, z_{0})\ominus \chi _{t}(t_{0}, z_{0})}{(-r)} = \chi _{tt}(t_{0}, z_{0}). \end{aligned}$$

According to Theorem 2.4 we have the following theorem

### Theorem 2.8

Let a continuous fuzzy function $\chi :[a,b]\times [c,d] \rightarrow R_{F},$ such that, $[\chi (x,y)]^\alpha = [{\underline{\chi }}^\alpha (x,y), {\overline{\chi }}^\alpha (x,y)]$, and $0\le \alpha \le 1$. Then for all $(x,y)\in [a,b]\times [c,d]$ we have

(i) If $[D_{x}\chi (x,y)]^\alpha$ is $(i)-$differentiable with respect to *x* then $D_{x}{\underline{\chi }}^\alpha (x,y), D_{x}{\overline{\chi }}^\alpha (x,y)$ are differentiable with respect to *x* and $\big [D_{x}\chi (x,y)\big ]^\alpha = \Big [D_{x}{\underline{\chi }}^\alpha (x,y), D_{x}{\overline{\chi }}^\alpha (x,y)\Big ].$
(ii) If $[D_{y}\chi (x,y)]^\alpha$ is $(i)-$differentiable with respect to *y* then $D_{y}{\underline{\chi }}^\alpha (x,y), D_{y}{\overline{\chi }}^\alpha (x,y)$ are differentiable with respect to *y* and $\big [D_{y}\chi (x,y)\big ]^\alpha = \Big [D_{y}{\underline{\chi }}^\alpha (x,y), D_{y}{\overline{\chi }}^\alpha (x,y)\Big ].$
(iii) If $[D_{x}\chi (x,y)]^\alpha$ is $(ii)-$differentiable with respect to *x* then $D_{x}{\underline{\chi }}^\alpha (x,y), D_{x}{\overline{\chi }}^\alpha (x,y)$ are differentiable with respect to *x* and $\big [D_{x}\chi (x,y)\big ]^\alpha = \Big [D_{x}{\overline{\chi }}^\alpha (x,y), D_{x}{\underline{\chi }}^\alpha (x,y)\Big ].$
(iv) If $[D_{}\chi (x,y)]^\alpha$ is $(ii)-$differentiable with respect to *y* then $D_{y}{\underline{\chi }}^\alpha (x,y), D_{y}{\overline{\chi }}^\alpha (x,y)$ are differentiable with respect to *y* and $\big [D_{y}\chi (x,y)\big ]^\alpha = \Big [D_{y}{\overline{\chi }}^\alpha (x,y), D_{y}{\underline{\chi }}^\alpha (x,y)\Big ].$

## Natural transform of second order partial derivative

The commonly used integral transform for the solutions of differential equations are Laplace and Sumudu transform. But natural transform easily converges to these transforms. This specialty of natural transform provided the opportunity to use it as a checker on these transforms. The applications of the natural transform method turn out well for solutions of differential equations. Therefore in the current work, we propose natural transform to study second order partial differential equations

### Theorem 3.1

Let fuzzy valued function $\big [\chi (t,y)\big ]^\alpha$ be continuous such that, $e^{-st} \big [\chi (ut,y)\big ]^\alpha$, $e^{-st} \big [\chi _{t}(ut,y)\big ]^\alpha$ and $e^{-st} \big [\chi _{tt}(ut,y)\big ]^\alpha$ exists and continuous. Moreover they are Riemann integrable on, $[0,\infty )$ then

(A) If $\big [\chi (t,y)\big ]^\alpha$ and $\big [\chi _t(t,y)\big ]^\alpha$ are $(i)-$differentiable, then
  $$\begin{aligned} {\mathcal {N}}\big [\chi _{tt}(t,y)\big ]^\alpha = \bigg [\frac{s^2}{u^2} {\widehat{R}}^\alpha (s, u, y) \ominus \frac{s}{u^2}\chi ^\alpha (0,y)\bigg ] \ominus \frac{1}{u}{\chi _t}^\alpha (0,y). \end{aligned}$$
(B) If $\big [\chi (t,y)\big ]^\alpha$ is $(i)-$differentiable and $\big [\chi _t(t,y)\big ]^\alpha$ is $(ii)-$differentiable, then
  $$\begin{aligned} {\mathcal {N}}\big [\chi _{tt}(t,y)\big ]^\alpha = \left(-\frac{1}{u}{\chi _t}^\alpha (0,y)\right)\ominus \bigg [\left(-\frac{s^2}{u^2} {\widehat{R}}^\alpha (s, u, y)\right) \ominus \left(-\frac{s}{u^2}\chi ^\alpha (0,y)\right)\bigg ]. \end{aligned}$$
(C) If $\big [\chi (t,y)\big ]^\alpha$ is $(ii)-$differentiable and $\big [\chi _t(t,y)\big ]^\alpha$ is $(i)-$differentiable, then
  $$\begin{aligned} {\mathcal {N}}\big [\chi _{tt}(t,y)\big ]^\alpha = \bigg [\left(- \frac{s}{u^2}\chi ^\alpha (0,y)\right) \ominus \left(-\frac{s^2}{u^2} {\widehat{R}}^\alpha (s, u, y)\right)\bigg ]\ominus \frac{1}{u}{\chi _t}^\alpha (0,y). \end{aligned}$$
(D) If, $\big [\chi (t,y)\big ]^\alpha$ and $\big [\chi _t(t,y)\big ]^\alpha$ are $(ii)-$differentiable, then
  $$\begin{aligned} {\mathcal {N}}\big [\chi _{tt}(t,y)\big ]^\alpha = \left(-\frac{1}{u}{\chi _t}^\alpha (0,y)\right)\ominus \bigg [\frac{s}{u^2}\chi ^\alpha (0,y) \ominus \frac{s^2}{u^2} {\widehat{R}}^\alpha (s, u, y)\bigg ]. \end{aligned}$$

### Proof

(A) Assume that $\big [\chi (t,y)\big ]^\alpha$ and $\big [\chi _t(t,y)\big ]^\alpha$ are $(i)-$differentiable, then
  3.1 $$\begin{aligned} {\mathcal {N}} \big [\chi _t(t,y)\big ]^\alpha= & {} \frac{s}{u}\big [ {\widehat{R}}^\alpha (s, u, y)\big ] \ominus \frac{1}{u}\chi ^\alpha (0,y), \ \ \ \hbox {and}\nonumber \\ {\mathcal {N}}\big [\chi _{tt}(t,y)\big ]^\alpha= & {} \frac{s}{u}\big [ {\mathcal {N}}\{{\chi _{t}}^\alpha (t,y)\}\big ] \ominus \frac{1}{u}{\chi _{t}}^\alpha (0,y), \ \ \hbox {Using}\, 3.1\, \hbox {this identity yields},\nonumber \\ {\mathcal {N}}\big [\chi _{tt}(t,y)\big ]^\alpha= & {} \frac{s}{u}\bigg [ \frac{s}{u}\big [ {\widehat{R}}^\alpha (s, u, y)\big ] \ominus \frac{1}{u}\chi ^\alpha (0,y) \bigg ] \ominus \frac{1}{u}{\chi _{t}}^\alpha (0,y),\nonumber \\ {\mathcal {N}}\big [\chi _{tt}(t,y)\big ]^\alpha= & {} \bigg [ \frac{s^2}{u^2} {\widehat{R}}^\alpha (s, u, y) \ominus \frac{s}{u^2}\chi ^\alpha (0,y) \bigg ] \ominus \frac{1}{u}{\chi _{t}}^\alpha (0,y). \end{aligned}$$
(B) If $\big [\chi (t,y)\big ]^\alpha$ and $\big [\chi _t(t,y)\big ]^\alpha$ are $(i)-$differentiable and $(ii)-$differentiable respectively, then
  3.2 $$\begin{aligned} {\mathcal {N}}\big [\chi _t(t,y)\big ]^\alpha= & {} \frac{s}{u}\big [ {\widehat{R}}^\alpha (s, u, y)\big ] \ominus \frac{1}{u}\chi ^\alpha (0,y) \ \ \ \hbox {and}\nonumber \\ {\mathcal {N}}\big [\chi _{tt}(t,y)\big ]^\alpha= & {} \left(- \frac{1}{u}{\chi _{t}}^\alpha (0,y)\right) \ominus \left(-\frac{s}{u}\big [ {\mathcal {N}}\{{\chi _{t}}^\alpha (t,y)\}\big ]\right), \ \ \hbox {Using}\, 3.2 \,\hbox {this identity yields}, \nonumber \\ {\mathcal {N}}\big [\chi _{tt}(t,y)\big ]^\alpha= & {} \left(-\frac{1}{u}{\chi _{t}}^\alpha (0,y)\right) \ominus \bigg [-\frac{s}{u} \bigg ( \frac{s}{u}\big [{\widehat{R}}^\alpha (s, u, y)\big ] \ominus \frac{1}{u}\chi ^\alpha (0,y)\bigg ) \bigg ],\nonumber \\ {\mathcal {N}}\big [\chi _{tt}(t,y)\big ]^\alpha= & {} \left(- \frac{1}{u}{\chi _{t}}^\alpha (0,y)\right) \ominus \bigg [ \left(-\frac{s^2}{u^2} {\widehat{R}}^\alpha (s, u, y)\right) \ominus \left(-\frac{s}{u^2}\chi ^\alpha (0,y)\right) \bigg ]. \end{aligned}$$
(C) If $\big [\chi (t,y)\big ]^\alpha$ and $\big [\chi _t(t,y)\big ]^\alpha$ are $(ii)-$differentiable and $(i)-$differentiable respectively, then
  3.3 $$\begin{aligned} {\mathcal {N}}\big [\chi _t(t,y)\big ]^\alpha= & {} \left(-\frac{1}{u}\chi ^\alpha (0,y)\right) \ominus \bigg (-\frac{s}{u}\big [ {\widehat{R}}^\alpha (s, u, y)\big ]\bigg ) \ \ \ \hbox {and}\nonumber \\ {\mathcal {N}}\big [\chi _{tt}(t,y)\big ]^\alpha= & {} \frac{s}{u}\left[ {\mathcal {N}}\{{\chi _{t}}^\alpha (t,y)\}\right] \ominus \frac{1}{u}\chi ^\alpha (0,y), \ \ \hbox {Using} \,3.3\, \hbox {this identity yields},\nonumber \\ {\mathcal {N}}\big [\chi _{tt}(t,y)\big ]^\alpha= & {} \left(-\frac{s}{u^2}{\chi _{t}}^\alpha (0,y)\right) \ominus \bigg (-\frac{s^2}{u^2}\big [{\widehat{R}}^\alpha (s, u, y)\big ]\bigg ) \ominus \frac{1}{u}\chi ^\alpha (0,y)\big ),\nonumber \\ {\mathcal {N}}\big [\chi _{tt}(t,y)\big ]^\alpha= & {} \bigg [\left(-\frac{1}{u}\chi ^\alpha (0,y)\right)\ominus \left(-\frac{s^2}{u^2} {\widehat{R}}^\alpha (s, u, y)\right)\bigg ]\ominus \frac{1}{u}{\chi _{t}}^\alpha (0,y). \end{aligned}$$
(D) If $\big [\chi (t,y)\big ]^\alpha$ and $\big [\chi _t(t,y)\big ]^\alpha$ are $(ii)-$differentiable, then
  3.4 $$\begin{aligned} {\mathcal {N}}\big [\chi _t(t,y)\big ]^\alpha= & {} \left(-\frac{1}{u}\chi ^\alpha (0,y)\right) \ominus \bigg (-\frac{s}{u}\big [ {\widehat{R}}^\alpha (s, u, y)\big ]\bigg ) \ \ \ \hbox {and}\nonumber \\ {\mathcal {N}}\big [\chi _{tt}(t,y)\big ]^\alpha= & {} \left(-\frac{1}{u}{\chi _{t}}^\alpha (0,y)\right) \ominus \left(-\frac{s}{u}\big [ {\mathcal {N}}\{{\chi _{t}}^\alpha (t,y)\}\big ]\right), \ \ \hbox {Using}\,3.4\, \hbox {this identity yields}, \nonumber \\ {\mathcal {N}}\big [\chi _{tt}(t,y)\big ]^\alpha= & {} \left(-\frac{1}{u}{\chi _{t}}^\alpha (0,y)\right) \ominus \bigg [-\frac{s}{u}\bigg (\left(-\frac{1}{u}{\chi _{t}}^\alpha (0,y)\right) \ominus \left(-\frac{s}{u}[{\widehat{R}}^\alpha (s, u, y)]\right)\bigg )\bigg ],\nonumber \\ {\mathcal {N}}\big [\chi _{tt}(t,y)\big ]^\alpha= & {} \left(-\frac{1}{u}{\chi _{t}}^\alpha (0,y)\right)\ominus \bigg [\frac{s}{u^2}\chi ^\alpha (0,y)\ominus \frac{s^2}{u^2} {\widehat{R}}^\alpha (s, u, y))\bigg ]. \end{aligned}$$

$$\square$$

### Theorem 3.2

Let the fuzzy function, $\chi :[0,\infty [\times [0,\infty [\rightarrow R_{F},$ be continuous and $e^{-st} \big [\chi _y(ut,y)\big ]^\alpha ,$ is improper fuzzy Riemann integrable in the interval, $[0,\infty ),$ then for natural transform, ${\mathcal {N}}\big [\chi _y(t,y)\big ]^\alpha ,$ with respect to *t*,  hold the following identity.

$$\begin{aligned} {\mathcal {N}}\Big \{\frac{\partial }{\partial y}\big [\chi (t,y)\big ]^\alpha \Big \} = \frac{\partial }{\partial y}{\mathcal {N}}\big [\chi (t,y)\big ]^\alpha . \end{aligned}$$

### Proof

The fuzzy Natural transform is define, as

$$\begin{aligned} {\mathcal {N}}\Big \{\frac{\partial }{\partial y}\big [\chi (t,y)\big ]^\alpha \Big \}= & {} \int _0^{\infty }e^{-st}\frac{\partial }{\partial y}\big [\chi (ut,y)\big ]^\alpha dt,\ \ \ \hbox {then}\\ {\mathcal {N}}\Big \{\frac{\partial }{\partial y}\chi (t,y)\Big \}= & {} \Big [ \int _0^{\infty }e^{-st}\frac{\partial }{\partial y}({\underline{\chi }}^\alpha (ut,y))dt, \int _0^{\infty }e^{-st}\frac{\partial }{\partial y}({\overline{\chi }}^\alpha (ut,y))dt\Big ], \\= & {} \Big [\frac{\partial }{\partial y} \int _0^{\infty }e^{-st}{\underline{\chi }}^\alpha (ut,y)dt, \frac{\partial }{\partial y}\int _0^{\infty }e^{-st}{\overline{\chi }}^\alpha (ut,y)dt\Big ], \\= & {} \frac{\partial }{\partial y} \Big [ \int _0^{\infty }e^{-st}{\underline{\chi }}^\alpha (ut,y)dt, \int _0^{\infty }e^{-st}{\overline{\chi }}^\alpha (ut,y)dt\Big ], \\= & {} \frac{\partial }{\partial y}{\mathcal {N}}\big [\chi (t,y)\big ]^\alpha . \end{aligned}$$

$$\square$$

## Fuzzy advection-diffusion equations with natural transform and adomian decomposition

First we discuss the following linear advection-diffusion fuzzy partial differential equations with constant diffusion coefficient $a>0$ and constant advection velocity *b*.

4.1 $$\begin{aligned} \left\{ \begin{aligned} \big [\chi _{y}(t,y)\big ]^\alpha= & {} a \big [\chi _{tt}(t,y)\big ]^\alpha \ominus b [\chi _{t}(t,y)]^\alpha ,\ \ \ \ \ \ \ \\ \chi ^\alpha (0,y)= & {} p_{0}(y,\alpha ) = ({\underline{p}}_{0}(y,\alpha ), {\overline{p}}_{0}(y,\alpha )),\\ {\chi _{t}}^\alpha (0,y)= & {} q_{0}(y,\alpha ) = ({\underline{q}}_{0}(y,\alpha ), {\overline{q}}_{0}(y,\alpha )),\\ \chi ^\alpha (t,0)= & {} r_{0}(t,\alpha ) = ({\underline{r}}_{0}(t,\alpha ), {\overline{r}}_{0}(t,\alpha )). \end{aligned}\right. \end{aligned}$$

Where, $p_{0}(y,\alpha ),$ $q_{0}(y,\alpha )$ and $r_{0}(t,\alpha )$ are the initial values and fuzzy function, $\chi :[0,\infty [\times [0,\infty [\rightarrow R_F,$ is define on $y,t\ge 0$ while $[g(t,y)]^\alpha$ is fuzzy function.

The fuzzy solutions of 4.1, can be discussed in the following four cases.

*Case 1*: The natural transform of following two cases are in the form

4.2 $$\begin{aligned} \left\{ \begin{aligned} {\mathcal {N}}\{{{\underline{\chi }}_{y}}^\alpha (t,y)\}= & {} a {\mathcal {N}}\{{{\underline{\chi }}_{tt}}^\alpha (t,y)\} \ominus b{\mathcal {N}}\{{{\underline{\chi }}_{t}}^\alpha (t,y)\},\\ {\mathcal {N}}\{{{\overline{\chi }}_{y}}^\alpha (t,y)\}= & {} a {\mathcal {N}}\{{{\overline{\chi }}_{tt}}^\alpha (t,y)\} \ominus b {\mathcal {N}}\{{{\overline{\chi }}_{t}}^\alpha (t,y)\}. \end{aligned}\right. \end{aligned}$$

The following two cases can be solved by using appropriate integrating factor.

*Case (a)*: Let $\frac{\partial }{\partial y}\big [\chi (t,y)\big ]^\alpha$, $\frac{\partial }{\partial t}\big [\chi (t,y)\big ]^\alpha$ and $\frac{\partial ^2}{\partial t^2}\big [\chi (t,y)\big ]^\alpha$ are $(i)-$differentiable (OR) $(ii)-$differentiable, then using Theorem (3.1), in Eq. (4.2), one can get

4.3 $$\begin{aligned} \left\{ \begin{aligned} {\mathcal {N}}\left\{\frac{\partial }{\partial y}{{\underline{\chi }}}^\alpha (t,y)\right\}= & {} \bigg [ \left( \frac{as^2}{u^2}- \frac{bs}{u}\right) \underline{{\widehat{R}}}^\alpha (s, u, y) \ominus \left(\frac{as}{u^2} - \frac{b}{u}\right){\underline{\chi }}^\alpha (0,y) \bigg ] \ominus \frac{a}{u}{{\underline{\chi }}_{t}}^\alpha (0,y),\\ {\mathcal {N}}\left\{\frac{\partial }{\partial y}{\overline{\chi }}^\alpha (t,y)\right\}= & {} \bigg [ \left( \frac{as^2}{u^2}- \frac{bs}{u}\right) {\widehat{{\overline{R}}}}^\alpha (s, u, y) \ominus \left(\frac{as}{u^2} - \frac{b}{u}\right){\overline{\chi }}^\alpha (0,y) \bigg ] \ominus \frac{a}{u}{{\overline{\chi }}_{t}}^\alpha (0,y). \end{aligned}\right. \end{aligned}$$

Using Theorem (3.2) and initial conditions in Eq. (4.3), one can get

4.4 $$\begin{aligned} \left\{ \begin{aligned} \frac{\partial }{\partial y}\underline{{\widehat{R}}}^\alpha (s, u, y)= & {} \bigg [ \left( \frac{as^2}{u^2}- \frac{bs}{u}\right) \underline{{\widehat{R}}}^\alpha (s, u, y) \ominus \left(\frac{as}{u^2} - \frac{b}{u}\right){\underline{\chi }}^\alpha (0,y) \bigg ] \ominus \frac{a}{u}{{\underline{\chi }}_{t}}^\alpha (0,y),\\ \frac{\partial }{\partial y}{\widehat{{\overline{R}}}^\alpha }(s, u, y)= & {} \bigg [ \left( \frac{as^2}{u^2}- \frac{bs}{u}\right) {\widehat{{\overline{R}}}}^\alpha (s, u, y) \ominus \left(\frac{as}{u^2} - \frac{b}{u}\right){\overline{\chi }}^\alpha (0,y) \bigg ] \ominus \frac{a}{u}{{\overline{\chi }}_{t}}^\alpha (0,y). \end{aligned}\right. \end{aligned}$$

Solving Eq. (4.4) satisfying the initial conditions, assume that solution is obtained as follows

4.5 $$\begin{aligned} \left\{ \begin{aligned} \underline{{\widehat{R}}}^\alpha (s, u, y)= & {} R_1(s,u,y,\alpha )\\ {\widehat{{\overline{R}}}}^\alpha (s, u, y)= & {} G_1(s,u,y,\alpha ). \end{aligned}\right. \end{aligned}$$

Taking inverse natural transform of Eq. (4.5) one can find ${\underline{\chi }}(t,y)$ and ${\overline{\chi }}(t,y),$ as follows.

4.6 $$\begin{aligned} \left\{ \begin{aligned} {\underline{\chi }}^\alpha (t,y)= & {} {\mathcal {N}}^{-1}\{R_1(s,u,y,\alpha )\},\\ {\overline{\chi }}^\alpha (t,y)= & {} {\mathcal {N}}^{-1}\{G_1(s,u,y,\alpha )\}. \end{aligned}\right. \end{aligned}$$

The solutions of Eq. (4.6) is valid only if ${\underline{\chi }}^\alpha (t,y)\le {\overline{\chi }}^\alpha (t,y),$ ${{\underline{\chi }}_{y}}^\alpha (t,y)\le {{\overline{\chi }}_{y}}^\alpha (t,y),$ ${{\underline{\chi }}_{t}}^\alpha (t,y)\le {{\overline{\chi }}_{t}}^\alpha (t,y)$ and ${{\underline{\chi }}_{tt}}^\alpha (t,y)\le {{\overline{\chi }}_{tt}}^\alpha (t,y).$

*Case (b)*: Let $\frac{\partial }{\partial y}\big [\chi (t,y)\big ]^\alpha$, $\frac{\partial }{\partial t}\big [\chi (t,y)\big ]^\alpha$ are $(i)-$differentiable and $\frac{\partial ^2}{\partial t^2}\big [\chi (t,y)\big ]^\alpha$ is $(ii)-$differentiable (OR) $\frac{\partial }{\partial y}\big [\chi (t,y)\big ]^\alpha$, $\frac{\partial }{\partial t}\big [\chi (t,y)\big ]^\alpha$ are $(ii)-$differentiable and $\frac{\partial ^2}{\partial t^2}\big [\chi (t,y)\big ]^\alpha$ is $(i)-$differentiable then using Theorem (3.1), in Eq.(4.2) one can get

4.7 $$\begin{aligned} \left\{ \begin{aligned} {\mathcal {N}}\left\{\frac{\partial }{\partial y}{{\underline{\chi }}}^\alpha (t,y)\right\}= & {} \left(- \frac{a}{u}{{\overline{\chi }}_{t}}^\alpha (0,y)\right) \ominus \bigg [\left (\frac{bs}{u}-\frac{as^2}{u^2}\right) \underline{{\widehat{R}}}^\alpha (s, u, y)) \ominus \left(\frac{b}{u}-\frac{as}{u^2}\right){\underline{\chi }}^\alpha (0,y)) \bigg ],\\ {\mathcal {N}}\left\{\frac{\partial }{\partial y}\overline{\chi }^\alpha (t,y)\right\}= & {} \left(- \frac{a}{u}{{\underline{\chi }}_{t}}^\alpha (0,y)\right) \ominus \bigg [ \left(\frac{bs}{u}-\frac{as^2}{u^2}\right) {\widehat{{\overline{R}}}}^\alpha (s, u, y) \ominus \left(\frac{b}{u}-\frac{as}{u^2}\right){\overline{\chi }}^\alpha (0,y) \bigg ]. \end{aligned}\right. \end{aligned}$$

Using Theorem (3.2) and initial conditions in Eq.(4.7), one can get

4.8 $$\begin{aligned} \left\{ \begin{aligned} \frac{\partial }{\partial y}\underline{{\widehat{R}}}^\alpha (s, u, y)= & {} \bigg [ \left(\frac{as^2}{u^2}-\frac{bs}{u}\right) \underline{{\widehat{R}}}^\alpha (s, u, y) \ominus \left(\frac{as}{u^2}-\frac{b}{u}\right){\underline{p}}_{0}(y,\alpha ) \bigg ] \ominus \frac{a}{u}{\overline{q}}_{0}(y,\alpha ),\\ \frac{\partial }{\partial y}\overline{{\widehat{R}}}^\alpha (s, u, y)= & {} \bigg [ \left(\frac{as^2}{u^2}-\frac{bs}{u}\right) {\widehat{{\overline{R}}}^\alpha }(s, u, y) \ominus \left(\frac{as}{u^2}-\frac{b}{u}\right){\overline{p}}_{0}(y,\alpha ) \bigg ] \ominus \frac{a}{u}{\underline{q}}_{0}(y,\alpha ). \end{aligned}\right. \end{aligned}$$

Solving Eq. (4.8) and using initial conditions, assume that solution is obtained as follows

4.9 $$\begin{aligned} \left\{ \begin{aligned} \underline{{\widehat{R}}}^\alpha (s, u, y)= & {} R_2(s,u,y,\alpha ),\\ {\widehat{{\overline{R}}}}^\alpha (s, u, y)= & {} G_2(s,u,y,\alpha ). \end{aligned}\right. \end{aligned}$$

Taking inverse natural transform of Eq. (4.9) one can get  ${\underline{\chi }}^\alpha (t,y)$ and ${\overline{\chi }}^\alpha (t,y),$ as follows

4.10 $$\begin{aligned} \left\{ \begin{aligned} {\underline{\chi }}^\alpha (t,y)= & {} {\mathcal {N}}^{-1}\{R_2(s,u,y,\alpha )\},\\ {\overline{\chi }}^\alpha (t,y)= & {} {\mathcal {N}}^{-1}\{G_2(s,u,y,\alpha )\}. \end{aligned}\right. \end{aligned}$$

The solutions of Eq. (4.10), is valid only if ${\underline{\chi }}^\alpha (t,y)\le {\overline{\chi }}^\alpha (t,y),$ ${{\underline{\chi }}_{y}}^\alpha (t,y)\le {{\overline{\chi }}_{y}}^\alpha (t,y),$ ${{\underline{\chi }}_{t}}^\alpha (t,y)\le {{\overline{\chi }}_{t}}^\alpha (t,y)$ and ${{\underline{\chi }}_{tt}}^\alpha (t,y)\le {{\overline{\chi }}_{tt}}^\alpha (t,y).$

*Case 2*: The natural transform of following two cases are in the form

4.11 $$\begin{aligned} \left\{ \begin{aligned} {\mathcal {N}}\{{{\underline{\chi }}_{y}}^\alpha (t,y)\}= & {} a {\mathcal {N}}\{{{\overline{\chi }}_{tt}}^\alpha (t,y)\} \oplus b {\mathcal {N}}\{{{\overline{\chi }}_{t}}^\alpha (t,y)\},\\ {\mathcal {N}}\{{{\overline{\chi }}_{y}}^\alpha (t,y)\}= & {} a {\mathcal {N}}\{{{\underline{\chi }}_{tt}}^\alpha (t,y)\} \oplus b {\mathcal {N}}\{{{\underline{\chi }}_{t}}^\alpha (t,y)\}. \end{aligned}\right. \end{aligned}$$

The right and left side of these equations have ${\underline{\chi }}^\alpha (t,y)$ and ${\overline{\chi }}^\alpha (t,y)$ therefore fuzzy solutions of the following two cases can be obtain by natural Adomian decomposition method.

*Case (a)*: Let $\frac{\partial }{\partial y}\big [\chi (t,y)\big ]^\alpha$ is $(i)-$differentiable and $\frac{\partial }{\partial t}\big [\chi (t,y)\big ]^\alpha$ and $\frac{\partial ^2}{\partial t^2}\big [\chi (t,y)\big ]^\alpha$ are $(ii)-$differentiable (OR) $\frac{\partial }{\partial y}\big [\chi (t,y)\big ]^\alpha$ is $(ii)-$differentiable and $\frac{\partial }{\partial t}\big [\chi (t,y)\big ]^\alpha$ and $\frac{\partial ^2}{\partial t^2}\big [\chi (t,y)\big ]^\alpha$ are $(i)-$differentiable then using Theorem (3.1) in Eq. (4.11), one can get

4.12 $$\begin{aligned} \left\{ \begin{aligned} {\mathcal {N}}\left\{\frac{\partial }{\partial y}{{\underline{\chi }}}^\alpha (t,y)\right\}= & {} -\frac{a}{u}{{\overline{\chi }}_{t}}^\alpha (0,y)\ominus \bigg [\left(\frac{as}{u^2}-\frac{b}{u}\right){\overline{\chi }}^\alpha (0,y)\ominus \left(\frac{as^2}{u^2}-\frac{bs}{u}\right) {\widehat{{\overline{R}}}^\alpha }(s, u, y)\bigg ],\\ {\mathcal {N}}\left\{\frac{\partial }{\partial y}\overline{\chi }^\alpha (t,y)\right\}= & {} -\frac{a}{u}{{\underline{\chi }}_{t}}^\alpha (0,y)\ominus \bigg [\left(\frac{as}{u^2}-\frac{b}{u}\right){\underline{\chi }}^\alpha (0,y)\ominus \left(\frac{as^2}{u^2}-\frac{bs}{u}\right) \underline{{\widehat{R}}}^\alpha (s, u, y)\bigg ]. \end{aligned}\right. \end{aligned}$$

Rearranged Eq. (4.12) for series solutions and using initial conditions, one can get

4.13 $$\begin{aligned} \left\{ \begin{aligned} \overline{{\widehat{R}}}^\alpha (s,u,y)= & {} \frac{1}{s}{\overline{p}}_{0}(y,\alpha ) \oplus \big (\frac{a}{b(s-\frac{bu}{a})}-\frac{a}{bs}\big ){\overline{q}}_{0}(y,\alpha ) \oplus \big (\frac{u^2}{as^2-bus}\big ) {\mathcal {N}}\{\frac{\partial }{\partial y}{{\underline{\chi }}}^\alpha (t,y)\},\\ \underline{{\widehat{R}}}^\alpha (s,u,y)= & {} \frac{1}{s}{\underline{p}}_{0}(y,\alpha ) \oplus \big (\frac{a}{b(s-\frac{bu}{a})}-\frac{a}{bs}\big ){\underline{q}}_{0}(y,\alpha ) \oplus \big (\frac{u^2}{as^2-bus}\big ) {\mathcal {N}}\{\frac{\partial }{\partial y}\overline{\chi }^\alpha (t,y)\}. \end{aligned}\right. \end{aligned}$$

Taking inverse natural transform of Eq. (4.13), one can get

4.14 $$\begin{aligned} \left\{ \begin{aligned} {\overline{\chi }}^\alpha (t,y)=\, & {} {\overline{p}}_{0}(y,\alpha ) \oplus \frac{a}{b} (e^{\frac{b}{a}t}-1){\overline{q}}_{0}(y,\alpha ) \oplus {\mathcal {N}}^{-1} \Big [\big (\frac{u}{b(s-\frac{bu}{a})}-\frac{u}{bs}\big ) {\mathcal {N}}\Big \{\frac{\partial }{\partial y}{{\underline{\chi }}}^\alpha (t,y)\Big \}\Big ],\\ {\underline{\chi }}^\alpha (t,y)=\, & {} {\underline{p}}_{0}(y,\alpha ) \oplus \frac{a}{b} (e^{\frac{b}{a}t}-1){\underline{q}}_{0}(y,\alpha ) \oplus {\mathcal {N}}^{-1} \Big [\big (\frac{u}{b(s-\frac{bu}{a})}-\frac{u}{bs}\big ) {\mathcal {N}}\Big \{\frac{\partial }{\partial y}\overline{\chi }^\alpha (t,y)\Big \}\Big ]. \end{aligned}\right. \end{aligned}$$

The right and left side of these equations have opposite cases ${\underline{\chi }}^\alpha (t,y)$ and ${\overline{\chi }}^\alpha (t,y)$ therefore we decompose it be Adomiam decomposition method to obtain recursive relations, with

4.15 $$\begin{aligned} \left\{ \begin{aligned} {{\overline{\chi }}_{0}}^\alpha (t,y)=\, & {} {\overline{p}}_{0}(y,\alpha ) \oplus \frac{a}{b} \left(e^{\frac{b}{a}t}-1\right){\overline{q}}_{0}(y,\alpha ),\\ {{\underline{\chi }}_{0}}^\alpha (t,y)=\, & {} {\underline{p}}_{0}(y,\alpha ) \oplus \frac{a}{b} \left(e^{\frac{b}{a}t}-1\right){\underline{q}}_{0}(y,\alpha ). \end{aligned}\right. \end{aligned}$$

4.16 $$\begin{aligned} \left\{ \begin{aligned} {{\overline{\chi }}_{n+1}}^\alpha (t,y)= \,& {} {\mathcal {N}}^{-1} \Bigg [\bigg (\frac{u}{b\left(s-\frac{bu}{a}\right)}-\frac{u}{bs}\bigg ) {\mathcal {N}}\Big \{\frac{\partial }{\partial y}{{{\underline{\chi }}}_{n}}^\alpha (t,y)\Big \}\Bigg ],\\ {{\underline{\chi }}_{n+1}}^\alpha (t,y)=\,& {} {\mathcal {N}}^{-1} \Bigg [\bigg (\frac{u}{b\left(s-\frac{bu}{a}\right)}-\frac{u}{bs}\bigg ) {\mathcal {N}}\Big \{\frac{\partial }{\partial y}{\overline{\chi }_{n}}^\alpha (t,y)\Big \}\Bigg ]. \end{aligned}\right. \end{aligned}$$

Evaluating, $\big [\chi _{0}\big ]^\alpha =\big [{{\underline{\chi }}_{0}}^\alpha , {{\overline{\chi }}_{0}}^\alpha \big ],$ $\big [\chi _{1}\big ]^\alpha =\big [{{\underline{\chi }}_{1}}^\alpha , {{\overline{\chi }}_{1}}^\alpha \big ],$ $\big [\chi _{2}\big ]^\alpha =\big [{{\underline{\chi }}_{2}}^\alpha , {{\overline{\chi }}_{2}}^\alpha \big ],$... the required solution is obtained, as

$$\begin{aligned} \left\{ \begin{aligned} {\underline{\chi }}^\alpha (t,y)=\, & {} {\sum _{n=0}^\infty } {{\underline{\chi }}_{n}}^\alpha (t,y),\\ {\overline{\chi }}^\alpha (t,y)= \,& {} {\sum _{n=0}^\infty } {{\overline{\chi }}_{n}}^\alpha (t,y). \end{aligned}\right. \end{aligned}$$

The series fuzzy solutions of Eq. (4.1), can be obtained, as

$$\begin{aligned} \big [\chi (t,y)\big ]^\alpha = \big [\chi _{0}(t,y)\big ]^\alpha + \big [\chi _{1}(t,y)\big ]^\alpha + \big [\chi _{2}(t,y)\big ]^\alpha +...+ \big [\chi _{n}(t,y)\big ]^\alpha \end{aligned}$$

The solutions is valid only if ${\underline{\chi }}^\alpha (t,y)\le {\overline{\chi }}^\alpha (t,y),$ ${{\underline{\chi }}_{y}}^\alpha (t,y)\le {\overline{\chi }}_{y}(t,y),$ ${{\underline{\chi }}_{t}}^\alpha (t,y)\le {{\overline{\chi }}_{t}}^\alpha (t,y)$ and ${{\underline{\chi }}_{tt}}^\alpha (t,y)\le {{\overline{\chi }}_{tt}}^\alpha (t,y).$

*Case (b)*: Let $\frac{\partial }{\partial y}\big [\chi (t,y)\big ]^\alpha$ and $\frac{\partial ^2}{\partial t^2}\big [\chi (t,y)\big ]^\alpha ,$ are $(i)-$differentiable and $\frac{\partial }{\partial t}\big [\chi (t,y)\big ]^\alpha$ is $(ii)-$differentiable (OR) $\frac{\partial }{\partial y}\chi ^\alpha (t,y)$ and $\frac{\partial ^2}{\partial t^2}\big [\chi (t,y)\big ]^\alpha$ are $(ii)-$differentiable and $\frac{\partial }{\partial t}\big [\chi (t,y)\big ]^\alpha$ is $(i)-$differentiable then using Theorem (3.1) in Eq. (4.11), one can get

4.17 $$\begin{aligned} \left\{ \begin{aligned} {\mathcal {N}}\{\frac{\partial }{\partial y}{{\underline{\chi }}}^\alpha (t,y)\}=\, & {} \bigg [\left(\frac{b}{u}-\frac{as}{u^2}\right){\overline{\chi }}^\alpha (0,y))\ominus \left(\frac{bs}{u}-\frac{as^2}{u^2}\right) {\widehat{{\overline{R}}}^\alpha }(s, u, y))\bigg ]\ominus \frac{a}{u}{{\underline{\chi }}_{t}}^\alpha (0,y),\\ {\mathcal {N}}\{\frac{\partial }{\partial y}\overline{\chi }^\alpha (t,y)\}= \,& {} \bigg [\left(\frac{b}{u}-\frac{as}{u^2}\right){\underline{\chi }}^\alpha (0,y))\ominus \left(\frac{bs}{u}-\frac{as^2}{u^2}\right) \underline{{\widehat{R}}}^\alpha (s, u, y))\bigg ]\ominus \frac{a}{u}{{\overline{\chi }}_{t}}^\alpha (0,y). \end{aligned}\right. \end{aligned}$$

Rearranged Eq. (4.17) for series solutions and using initial conditions, one can get

4.18 $$\begin{aligned} \left\{ \begin{aligned} {\overline{\chi }}^\alpha (s,y)= \,& {} \frac{1}{s}{\overline{p}}_{0}(y,\alpha ) \oplus \frac{a}{b}\big (\frac{1}{s-\frac{u}{a})} -\frac{1}{s}\big ){\underline{q}}_{0}(y,\alpha ) \oplus \big (\frac{u^2}{as^2-bus}\big ) {\mathcal {N}}\{\frac{\partial }{\partial y}{{\underline{\chi }}}^\alpha (t,y)\},\\ {\underline{\chi }}^\alpha (s,y)=\, & {} \frac{1}{s}{\underline{p}}_{0}(y,\alpha ) \oplus \frac{a}{b}\big (\frac{1}{s-\frac{u}{a})} -\frac{1}{s}\big ){\overline{q}}_{0}(y,\alpha ) \oplus \big (\frac{u^2}{as^2-bus}\big ) {\mathcal {N}}\{\frac{\partial }{\partial y}\overline{\chi }^\alpha (t,y)\}. \end{aligned}\right. \end{aligned}$$

Taking inverse natural transform of Eq. (4.18), one can get

4.19 $$\begin{aligned} \left\{ \begin{aligned} {\overline{\chi }}^\alpha (t,y)=\, & {} {\overline{p}}_{0}(y,\alpha ) \oplus \frac{a}{b} ( e^{\frac{b}{a}t}-1){\underline{q}}_{0}(y,\alpha ) \oplus {\mathcal {N}}^{-1} \Big [\big (\frac{u}{b(s-\frac{bu}{a})}-\frac{u}{bs}\big ) {\mathcal {N}}\Big \{\frac{\partial }{\partial y}{{\underline{\chi }}}^\alpha (t,y)\Big \}\Big ],\\ {\underline{\chi }}^\alpha (t,y)=\, & {} {\underline{p}}_{0}(y,\alpha ) \oplus \frac{a}{b} (e^{\frac{b}{a}t}-1){\overline{q}}_{0}(y,\alpha ) \oplus {\mathcal {N}}^{-1} \Big [\big (\frac{u}{b(s-\frac{bu}{a})}-\frac{u}{bs}\big ) {\mathcal {N}}\Big \{\frac{\partial }{\partial y}\overline{\chi }^\alpha (t,y)\Big \}\Big ]. \end{aligned}\right. \end{aligned}$$

The right and left side of these equations have opposite cases ${\underline{\chi }}^\alpha (t,y)$ and ${\overline{\chi }}^\alpha (t,y)$ therefore we decompose it with Adomiam decomposition method to obtain recursive relations, with

4.20 $$\begin{aligned} \left\{ \begin{aligned} {{\overline{\chi }}_{0}}^\alpha (t,y)= \,& {} {\overline{p}}_{0}(y,\alpha ) \oplus \frac{a}{b} (e^{\frac{b}{a}t}-1){\underline{q}}_{0}(y,\alpha ),\\ {{\underline{\chi }}_{0}}^\alpha (t,y)= \,& {} {\underline{p}}_{0}(y,\alpha ) \oplus \frac{a}{b} (e^{\frac{b}{a}t}-1){\overline{q}}_{0}(y,\alpha ). \end{aligned}\right. \end{aligned}$$

4.21 $$\begin{aligned} \left\{ \begin{aligned} {{\underline{\chi }}_{n+1}}^\alpha (t,y)=\, & {} {\mathcal {N}}^{-1} \Big [\big (\frac{u}{b(s-\frac{bu}{a})}-\frac{u}{bs}\big ) {\mathcal {N}}\Big \{\frac{\partial }{\partial y}{\overline{\chi }_{n}}^\alpha (t,y)\Big \}\Big ],\\ {{\overline{\chi }}_{n+1}}^\alpha (t,y)= \,& {} {\mathcal {N}}^{-1} \Big [\big (\frac{u}{b(s-\frac{bu}{a})}-\frac{u}{bs}\big ) {\mathcal {N}}\Big \{\frac{\partial }{\partial y}{{{\underline{\chi }}}_{n}}^\alpha (t,y)\Big \}\Big ]. \end{aligned}\right. \end{aligned}$$

Evaluating, $\big [\chi _{0}\big ]^\alpha =\big [{{\underline{\chi }}_{0}}^\alpha , {{\overline{\chi }}_{0}}^\alpha \big ],$ $\big [\chi _{1}\big ]^\alpha =\big [{{\underline{\chi }}_{1}}^\alpha , {{\overline{\chi }}_{1}}^\alpha \big ],$ $\big [\chi _{2}\big ]^\alpha =\big [{{\underline{\chi }}_{2}}^\alpha , {{\overline{\chi }}_{2}}^\alpha \big ],$... the required solution is obtained, as

$$\begin{aligned} \left\{ \begin{aligned} {\underline{\chi }}^\alpha (t,y)= & {} {\sum _{n=0}^\infty } {{\underline{\chi }}_{n}}^\alpha (t,y),\\ {\overline{\chi }}^\alpha (t,y)= & {} {\sum _{n=0}^\infty } {{\overline{\chi }}_{n}}^\alpha (t,y). \end{aligned}\right. \end{aligned}$$

The series fuzzy solutions of Eq. (4.1), can be obtained, as

$$\begin{aligned} \big [\chi (t,y)\big ]^\alpha = \big [\chi _{0}(t,y)\big ]^\alpha + \big [\chi _{1}(t,y)\big ]^\alpha + \big [\chi _{2}(t,y)\big ]^\alpha +...+ \big [\chi _{n}(t,y)\big ]^\alpha \end{aligned}$$

The solutions is valid only if ${\underline{\chi }}^\alpha (t,y)\le {\overline{\chi }}^\alpha (t,y),$ ${{\underline{\chi }}_{y}}^\alpha (t,y)\le {\overline{\chi }}_{y}(t,y),$ ${{\underline{\chi }}_{t}}^\alpha (t,y)\le {{\overline{\chi }}_{t}}^\alpha (t,y)$ and ${{\underline{\chi }}_{tt}}^\alpha (t,y)\le {{\overline{\chi }}_{tt}}^\alpha (t,y).$

### Example 4.1

Here we discuss the following linear advection-diffusion fuzzy partial differential equations.

4.22 $$\begin{aligned} \left\{ \begin{aligned} \big [\chi _{y}(t,y)\big ]^\alpha=\, & {} 2\big [\chi _{tt}(t,y)\big ]^\alpha \ominus \big [\chi _{y}(t,y)\big ]^\alpha , \\ \chi ^\alpha (0,y)=\, & {} p_{0}(y,\alpha ) = e^y(\alpha -1, 1-\alpha ),\\ {\chi _{t}}^\alpha (0,y)=\, & {} q_{0}(y,\alpha ) = e^y(\alpha -1, 1-\alpha ), \\ \chi ^\alpha (t,0)= & {} r_{0}(t,\alpha ) = e^t(\alpha -1, 1-\alpha ). \end{aligned}\right. \end{aligned}$$

Where, $$y\ge 0$$ and $$0\le t < 2.$$

*Case (1)*: For the fuzzy solutions the following two cases can be solved by using appropriate integrating factor.

*Case (a)*: Let $\frac{\partial }{\partial y}\big [\chi (t,y)\big ]^\alpha$, $\frac{\partial }{\partial t}\big [\chi (t,y)\big ]^\alpha$ and $\frac{\partial ^2}{\partial t^2}\big [\chi (t,y)\big ]^\alpha$ are $(i)-$differentiable (OR) $(ii)-$differentiable then substituting, $a=2, b=1$ and initial condition in Eq. (4.4), one can get

4.23 $$\begin{aligned} \left\{ \begin{aligned} \frac{\partial }{\partial y}\underline{{\widehat{R}}}^\alpha (s, u, y)= & {} \bigg [ \left( \frac{2s^2}{u^2}- \frac{s}{u}\right) \underline{{\widehat{R}}}^\alpha (s, u, y) \ominus \left(\frac{as}{u^2} - \frac{b}{u}\right){\underline{\chi }}^\alpha (0,y) \bigg ] \ominus \frac{2}{u}{{\underline{\chi }}_{t}}^\alpha (0,y),\\ \frac{\partial }{\partial y}{\widehat{{\overline{R}}}^\alpha }(s, u, y)= & {} \bigg [ \left( \frac{2s^2}{u^2}- \frac{s}{u}\right) {\widehat{{\overline{R}}}}^\alpha (s, u, y) \ominus \left(\frac{2s}{u^2} - \frac{1}{u}\right){\overline{\chi }}^\alpha (0,y) \bigg ] \ominus \frac{2}{u}{{\overline{\chi }}_{t}}^\alpha (0,y). \end{aligned}\right. \end{aligned}$$

Rearranged Eq. (4.23) and using initial conditions, one can get

4.24 $$\begin{aligned} \left\{ \begin{aligned} \frac{\partial }{\partial y}\underline{{\widehat{R}}}^\alpha (s, u, y) - \frac{2s^2-us}{u^2} \underline{{\widehat{R}}}^\alpha (s, u, y)= & {} \ominus \left (\frac{2s-u}{u^2}\right) e^y(\alpha -1) \ominus \frac{2}{u}e^y(\alpha -1),\\ \frac{\partial }{\partial y}{\widehat{{\overline{R}}}^\alpha }(s, u, y) - \frac{2s^2-us}{u^2} {\widehat{{\overline{R}}}}^\alpha (s, u, y)= & {} \ominus \ \frac{2s-u}{u^2}e^y(1-\alpha ) \ominus \frac{2}{u}e^y(1-\alpha ). \end{aligned}\right. \end{aligned}$$

Solving Eq. (4.24), by using integrating factor $I.F = e^{-\frac{2s^2-us}{u^2}y}$ and initial condition

$\big [\chi (t,0)\big ]^\alpha = e^{t}(\alpha -1,1-\alpha ),$ one can easily obtain the fuzzy solutions, $$\begin{aligned} \left\{ \begin{aligned} {\underline{\chi }}^\alpha (t,y)= & {} \ e^{t+y}(\alpha -1),\\ {\overline{\chi }}^\alpha (t,y)= & {} \ e^{t+y}(1-\alpha ). \end{aligned}\right. \end{aligned}$$

*Case (b)*: Let $\frac{\partial }{\partial y}\big [\chi (t,y)\big ]^\alpha$, $\frac{\partial }{\partial t}\big [\chi (t,y)\big ]^\alpha$ are $(i)-$differentiable and $\frac{\partial ^2}{\partial t^2}\big [\chi (t,y)\big ]^\alpha$ is $(ii)-$differentiable (OR) $\frac{\partial }{\partial y}\big [\chi (t,y)\big ]^\alpha$, $\frac{\partial }{\partial t}\big [\chi (t,y)\big ]^\alpha$ are $(ii)-$differentiable and $\frac{\partial ^2}{\partial t^2}\big [\chi (t,y)\big ]^\alpha$ is $(i)-$differentiable then substituting $a=2, b=1$ and initial condition in Eq. (4.8), one can get

4.25 $$\begin{aligned} \left\{ \begin{aligned} \frac{\partial }{\partial y}\underline{{\widehat{R}}}^\alpha (s, u, y)= & {} \bigg [ \left(\frac{as^2}{u^2}-\frac{bs}{u^2}\right) \underline{{\widehat{R}}}^\alpha (s, u, y) \ominus \left(\frac{as}{u^2}-\frac{b}{u}\right){\underline{p}}_{0}(y,\alpha ) \bigg ] \ominus \frac{a}{u}{\overline{q}}_{0}(y,\alpha ),\\ \frac{\partial }{\partial y}\overline{{\widehat{R}}}^\alpha (s, u, y)= & {} \bigg [ \left(\frac{as^2}{u^2}-\frac{bs}{u^2}\right) {\widehat{{\overline{R}}}^\alpha }(s, u, y) \ominus \left(\frac{as}{u^2}-\frac{b}{u}\right){\overline{p}}_{0}(y,\alpha ) \bigg ] \ominus \frac{a}{u}{\underline{q}}_{0}(y,\alpha ). \end{aligned}\right. \end{aligned}$$

Rearranged Eq. (4.25) and using initial conditions one can get

4.26 $$\begin{aligned} \left\{ \begin{aligned} \frac{\partial }{\partial y}\underline{{\widehat{R}}}^\alpha (s, u, y) - \frac{2s^2-us}{u^2} \underline{{\widehat{R}}}^\alpha (s, u, y)= & {} \ominus \ \frac{2s-u}{u^2} e^y(\alpha -1) \ominus \frac{2}{u}e^y(1-\alpha ),\\ \frac{\partial }{\partial y}{\widehat{{\overline{R}}}^\alpha }(s, u, y) - \frac{2s^2-us}{u^2} {\widehat{{\overline{R}}}}^\alpha (s, u, y)= & {} \ominus \ \frac{2s-u}{u^2}e^y(1-\alpha ) \ominus \frac{2}{u}e^y(\alpha -1). \end{aligned}\right. \end{aligned}$$

Solving Eq. (4.26), by using integrating factor $I.F = e^{-\frac{2s^2-us}{u^2}y},$

4.27 $$\begin{aligned} \left\{ \begin{aligned} \underline{{\widehat{R}}}^\alpha (s, u, y)= & {} \left(- \frac{2}{3\left(s +\frac{u}{2}\right)} + \frac{2}{3(s + u)}\right) e^y(\alpha -1) \oplus (\frac{2}{3(s +\frac{u}{2})} + \frac{1}{3(s + u)})e^y(1-\alpha ),\\ {{\overline{R}}}^\alpha (s, u, y)= & {} \left(- \frac{2}{3(s +\frac{u}{2})} + \frac{2}{3(s - u)}\right)e^y(1-\alpha ) \oplus \left(\frac{2}{3(s +\frac{u}{2})} + \frac{1}{3(s - u)}\right)e^y(\alpha -1). \end{aligned}\right. \end{aligned}$$

Take inverse natural transform and using initial condition, $\big [\chi (t,0)\big ]^\alpha = e^t (\alpha -1,1-\alpha ),$ one can easily obtain fuzzy solutions,

4.28 $$\begin{aligned} \left\{ \begin{aligned} {\underline{\chi }}^\alpha (t, y)= & {} \left(- \frac{2}{3}e^{-\frac{t}{2}+y} + \frac{2}{3}e^{t+y}\right) (\alpha -1) \oplus \left(\frac{2}{3}e^{-\frac{t}{2}+y} + \frac{1}{3}e^{t+y}\right)(1-\alpha ),\\ {\overline{\chi }}^\alpha (t, y)= & {} \left(- \frac{2}{3}e^{-\frac{t}{2}+y} + \frac{2}{3}e^{t+y}\right)(1-\alpha ) \oplus \left(\frac{2}{3}e^{-\frac{t}{2}+y} + \frac{1}{3}e^{t+y}\right)(\alpha -1). \end{aligned}\right. \end{aligned}$$

*Case (2)*: The following two cases are difficult to solved by integrating factor therefore, we apply Adomiam decomposition method.

*Case (a)*: Let $\frac{\partial }{\partial y}\big [\chi (t,y)\big ]^\alpha$ is $(i)-$differentiable and $\frac{\partial }{\partial t}\big [\chi (t,y)\big ]^\alpha$ and $\frac{\partial ^2}{\partial t^2}\big [\chi (t,y)\big ]^\alpha$ are $(ii)-$differentiable (OR) $\frac{\partial }{\partial y}\big [\chi (t,y)\big ]^\alpha$ is $(ii)-$differentiable and $\frac{\partial }{\partial t}\big [\chi (t,y)\big ]^\alpha$ and $\frac{\partial ^2}{\partial t^2}\big [\chi (t,y)\big ]^\alpha$ are $(i)-$differentiable. Using Eq. (4.15) and Eq. (4.16) with $a=2, b=1$ and initial conditions, one can get the following series

4.29 $$\begin{aligned} \left\{ \begin{aligned} {{\overline{\chi }}_{0}}^\alpha (t,y)= & {} (2e^{\frac{t}{2}}-1)e^{y}(1-\alpha ),\\ {{\underline{\chi }}_{0}}^\alpha (t,y)= & {} (2e^{\frac{t}{2}}-1)e^{y}(\alpha -1). \end{aligned}\right. \end{aligned}$$

4.30 $$\begin{aligned} \left\{ \begin{aligned} {{\overline{\chi }}_{n+1}}^\alpha (t,y)= & {} {\mathcal {N}}^{-1} \Big [\big (\frac{u}{s-\frac{u}{2}}-\frac{u}{s}\big )^{n}(\frac{2}{s-\frac{u}{2}}-\frac{1}{s})\Big \}\Big ]e^{y}(1-\alpha ),\\ {{\underline{\chi }}_{n+1}}^\alpha (t,y)= & {} {\mathcal {N}}^{-1} \Big [\big (\frac{u}{s-\frac{u}{2}}-\frac{u}{s}\big )^{n}(\frac{2}{s-\frac{u}{2}}-\frac{1}{s})\Big \}\Big ]e^{y}(\alpha -1). \end{aligned}\right. \end{aligned}$$

The series solution is obtained as follow

$$\begin{aligned} \big [\chi (t,y)\big ]^\alpha = \big [\chi _{0}(t,y)\big ]^\alpha + \big [\chi _{1}(t,y)\big ]^\alpha + \big [\chi _{2}(t,y)\big ]^\alpha + \big [\chi _{3}(t,y)\big ]^\alpha ... \end{aligned}$$

The series of Eq. (4.30) is infinite geometric series, therefore solution is obtained as follow

$$\begin{aligned} \left\{ \begin{aligned} {\underline{\chi }}^\alpha (t,y)= & {} {\mathcal {N}}^{-1}\left(\frac{2}{s-\frac{u}{2}}-\frac{1}{s}\right)\Bigg \{1+ \bigg (\frac{u}{s-\frac{u}{2}}-\frac{u}{s}\bigg )+ \bigg (\frac{u}{s-\frac{u}{2}}-\frac{u}{s}\bigg )^2 +...\Bigg \}e^{y}(1-\alpha )= e^{t+y}(\alpha -1),\\ {\overline{\chi }}^\alpha (t,y)= & {} {\mathcal {N}}^{-1}\left(\frac{2}{s-\frac{u}{2}}-\frac{1}{s}\right)\Bigg \{1+ \bigg (\frac{u}{s-\frac{u}{2}}-\frac{u}{s}\bigg )+ \bigg (\frac{u}{s-\frac{u}{2}}-\frac{u}{s}\bigg )^2 +...\Bigg \}e^{y}(1-\alpha )= e^{t+y}(1-\alpha ). \end{aligned}\right. \end{aligned}$$

*Case (b)*: Let $\frac{\partial }{\partial y}\big [\chi (t,y)\big ]^\alpha$ and $\frac{\partial ^2}{\partial t^2}\big [\chi (t,y)\big ]^\alpha$ are $(i)-$differentiable and $\frac{\partial }{\partial t}\big [\chi (t,y)\big ]^\alpha$ is $(ii)-$differentiable (OR) $\frac{\partial }{\partial y}\big [\chi (t,y)\big ]^\alpha$ and $\frac{\partial ^2}{\partial t^2}\big [\chi (t,y)\big ]^\alpha$ are $(ii)-$differentiable and $\frac{\partial }{\partial t}\big [\chi (t,y)\big ]^\alpha$ is $(i)-$differentiable. Using Eq. (4.20) and Eq. (4.21) with $a=2, b=1$ and initial conditions, one can get the following series

4.31 $$\begin{aligned} \left\{ \begin{aligned} {{\overline{\chi }}_{0}}^\alpha (t,y)= & {} e^{y}(1-\alpha ) \oplus 2(e^{\frac{t}{2}}-1)e^{y}(\alpha -1),\\ {{\underline{\chi }}_{0}}^\alpha (t,y)= & {} e^{y}(\alpha -1) \oplus (2e^{\frac{t}{2}}-1)e^{y}(1-\alpha ). \end{aligned}\right. \end{aligned}$$

4.32 $$\begin{aligned} \left\{ \begin{aligned} {{\overline{\chi }}_{n+1}}^\alpha (t,y)= & {} {\mathcal {N}}^{-1} \Big [\frac{1}{s} \big (\frac{u}{s-\frac{u}{2}}-\frac{u}{s}\big )^{n}\Big ]e^{y}(1-\alpha ) \oplus \Big [\big (\frac{u}{s-\frac{u}{2}}-\frac{u}{s}\big )^{n}2(\frac{1}{s-\frac{u}{2}}-\frac{1}{s})\Big ]e^{y}(\alpha -1),\\ {{\underline{\chi }}_{n+1}}^\alpha (t,y)= & {} {\mathcal {N}}^{-1} \Big [\frac{1}{s} \big (\frac{u}{s-\frac{u}{2}}-\frac{u}{s}\big )^{n}\Big \}\Big ]e^{y}(\alpha -1) \oplus \Big [\big (\frac{u}{s-\frac{u}{2}}-\frac{u}{s}\big )^{n}(\frac{2}{s-\frac{u}{2}}-\frac{1}{s})\Big ]e^{y}(1-\alpha ). \end{aligned}\right. \end{aligned}$$

The series of Eq. (4.32) is infinite geometric series, therefore solution is obtained as follow

$$\begin{aligned}{} & {} \left\{ \begin{aligned} {\underline{\chi }}^\alpha (t,y) = {\mathcal {N}}^{-1}\bigg(\frac{1}{s} \Bigg \{1+ \bigg (\frac{u}{s-\frac{u}{2}}-\frac{u}{s}\bigg )+ \bigg (\frac{u}{s-\frac{u}{2}} -\frac{u}{s}\bigg )^2 +...\Bigg \}e^{y}(\alpha -1)\\ \oplus \ {\mathcal {N}}^{-1}\left(\frac{2}{s-\frac{u}{2}} -\frac{1}{s}\right)\Bigg \{1+ \bigg (\frac{u}{s-\frac{u}{2}}-\frac{u}{s}\bigg )+ \bigg (\frac{u}{s-\frac{u}{2}} -\frac{u}{s}\bigg )^2 +...\Bigg \}e^{y}(1-\alpha )\bigg),\\ {\overline{\chi }}^\alpha (t,y) = {\mathcal {N}}^{-1}\bigg(\frac{1}{s}\Bigg \{1+ \bigg (\frac{u}{s-\frac{u}{2}}-\frac{u}{s}\bigg )+ \bigg (\frac{u}{s-\frac{u}{2}}-\frac{u}{s}\bigg )^2 +...\Bigg \}e^{y}(1-\alpha )\\ \oplus \ {\mathcal {N}}^{-1}\left(\frac{2}{s-\frac{u}{2}}-\frac{1}{s}\right)\Bigg \{1+ \bigg (\frac{u}{s-\frac{u}{2}}-\frac{u}{s}\bigg )+ \bigg (\frac{u}{s-\frac{u}{2}}-\frac{u}{s}\bigg )^2 +...\Bigg \}e^{y}(\alpha -1)\bigg). \end{aligned}\right. \\{} & {} \left\{ \begin{aligned} {\underline{\chi }}^\alpha (t, y)= & {} \bigg(- \frac{2}{3}e^{-\frac{t}{2}+y} + \frac{2}{3}e^{t+y}\bigg) (\alpha -1) \oplus \left(\frac{2}{3}e^{-\frac{t}{2}+y} + \frac{1}{3}e^{t+y}\right)(1-\alpha ),\\ {\overline{\chi }}^\alpha (t, y)= & {} \left(- \frac{2}{3}e^{-\frac{t}{2}+y} + \frac{2}{3}e^{t+y}\right)(1-\alpha ) \oplus \left(\frac{2}{3}e^{-\frac{t}{2}+y} + \frac{1}{3}e^{t+y}\right)(\alpha -1). \end{aligned}\right. \end{aligned}$$

## Fuzzy heat equations with natural transform and adomian decomposition

Now we have to discuss the solutions of fuzzy heat equations with the help of natural Adomian decomposition method.

5.1 $$\begin{aligned} \left\{ \begin{aligned} \big [\chi _{y}(t,y)\big ]^\alpha= & {} \big [\chi _{tt}(t,y)\big ]^\alpha \oplus [g(t,y)]^\alpha ,\ \ \ \ \ \ \ \\ \chi ^\alpha (0,y)= & {} p_{0}(y,\alpha ) = ({\underline{p}}_{0}(y,\alpha ), {\overline{p}}_{0}(y,\alpha )),\\ {\chi _{t}}^\alpha (0,y)= & {} q_{0}(y,\alpha ) = ({\underline{q}}_{0}(y,\alpha ), {\overline{q}}_{0}(y,\alpha )),\\ \chi ^\alpha (t,0)= & {} r_{0}(t,\alpha ) = ({\underline{r}}_{0}(t,\alpha ), {\overline{r}}_{0}(t,\alpha )). \end{aligned}\right. \end{aligned}$$

Where, $p_{0}(y,\alpha ),$ $q_{0}(y,\alpha )$ and $r_{0}(t,\alpha )$ are initial values and fuzzy function, $\chi :[0,\infty [\times [0,\infty [\rightarrow R_F,$ is define on $y,t\ge 0$ while $[g(t,y)]^\alpha$ is fuzzy function.

The fuzzy solutions of Eq (5.1), can be discussed in the following four cases.

*Case 1*: The natural transform of following two cases are in the form

5.2 $$\begin{aligned} \left\{ \begin{aligned} {\mathcal {N}}\{{{\underline{\chi }}_{y}}^\alpha (t,y)\}= & {} {\mathcal {N}}\{{{\underline{\chi }}_{tt}}^\alpha (t,y)\} \oplus {\mathcal {N}}\{{\underline{g}}^\alpha (t,y)\},\\ {\mathcal {N}}\{{{\overline{\chi }}_{y}}^\alpha (t,y)\}= & {} {\mathcal {N}}\{{{\overline{\chi }}_{tt}}^\alpha (t,y)\} \oplus {\mathcal {N}}\{{\overline{g}}^\alpha (t,y)\}. \end{aligned}\right. \end{aligned}$$

The following two cases can be solved by using appropriate integrating factor.

*Case (a)*: Let $\frac{\partial }{\partial y}\big [\chi (t,y)\big ]^\alpha$, $\frac{\partial }{\partial t}\big [\chi (t,y)\big ]^\alpha$ and $\frac{\partial ^2}{\partial t^2}\big [\chi (t,y)\big ]^\alpha$ are $(i)-$differentiable (OR) $(ii)-$differentiable, then using, Theorem (3.1), in Eq. (5.2), one can get

5.3 $$\begin{aligned} \left\{ \begin{aligned} {\mathcal {N}}\{\frac{\partial }{\partial y}{{\underline{\chi }}}^\alpha (t,y)\}= & {} \bigg [ \frac{s^2}{u^2} \underline{{\widehat{R}}}^\alpha (s, u, y) \ominus \frac{s}{u^2}{\underline{\chi }}^\alpha (0,y) \bigg ] \ominus \frac{1}{u}{{\underline{\chi }}_{t}}^\alpha (0,y) \oplus {\mathcal {N}}\{{\underline{g}}^\alpha (t,y)\},\\ {\mathcal {N}}\{\frac{\partial }{\partial y}{\overline{\chi }}^\alpha (t,y)\}= & {} \bigg [ \frac{s^2}{u^2} {\widehat{{\overline{R}}}}^\alpha (s, u, y) \ominus \frac{s}{u^2}{\overline{\chi }}^\alpha (0,y) \bigg ] \ominus \frac{1}{u}{{\overline{\chi }}_{t}}^\alpha (0,y) \oplus {\mathcal {N}}\{{\overline{g}}^\alpha (t,y)\}. \end{aligned}\right. \end{aligned}$$

Using Theorem (3.2) and initial conditions in Eq. (5.3), one can get

5.4 $$\begin{aligned} \left\{ \begin{aligned} \frac{\partial }{\partial y}\underline{{\widehat{R}}}^\alpha (s, u, y)= & {} \bigg [ \frac{s^2}{u^2} \underline{{\widehat{R}}}^\alpha (s, u, y) \ominus \frac{s}{u^2}{\underline{p}}_{0}(y,\alpha ) \bigg ] \ominus \frac{1}{u}{\underline{q}}_{0}(y,\alpha ) \oplus {\mathcal {N}}\{{{\underline{g}}}^\alpha (t,y)\},\\ \frac{\partial }{\partial y}{\widehat{{\overline{R}}}^\alpha }(s, u, y)= & {} \bigg [ \frac{s^2}{u^2} {\widehat{{\overline{R}}}}^\alpha (s, u, y) \ominus \frac{s}{u^2}{\overline{p}}_{0}(y,\alpha ) \bigg ] \ominus \frac{1}{u}{\overline{q}}_{0}(y,\alpha ) \oplus {\mathcal {N}}\{{\overline{g}}^\alpha (t,y)\}. \end{aligned}\right. \end{aligned}$$

Solving Eq. (5.4) satisfying the initial conditions, assume that solution is obtained as follows

5.5 $$\begin{aligned} \left\{ \begin{aligned} \underline{{\widehat{R}}}^\alpha (s, u, y)= & {} R_1(s,u,y,\alpha )\\ {\widehat{{\overline{R}}}}^\alpha (s, u, y)= & {} G_1(s,u,y,\alpha ). \end{aligned}\right. \end{aligned}$$

Taking inverse natural transform of Eq. (5.5) one can find ${\underline{\chi }}(t,y)$ and ${\overline{\chi }}(t,y),$ as follows.

5.6 $$\begin{aligned} \left\{ \begin{aligned} {\underline{\chi }}^\alpha (t,y)= & {} {\mathcal {N}}^{-1}\{R_1(s,u,y,\alpha )\},\\ {\overline{\chi }}^\alpha (t,y)= & {} {\mathcal {N}}^{-1}\{G_1(s,u,y,\alpha )\}. \end{aligned}\right. \end{aligned}$$

The solutions of Eq. (5.6) is valid only if ${\underline{\chi }}^\alpha (t,y)\le {\overline{\chi }}^\alpha (t,y),$ ${{\underline{\chi }}_{y}}^\alpha (t,y)\le {{\overline{\chi }}_{y}}^\alpha (t,y),$ ${{\underline{\chi }}_{t}}^\alpha (t,y)\le {{\overline{\chi }}_{t}}^\alpha (t,y)$ and ${{\underline{\chi }}_{tt}}^\alpha (t,y)\le {{\overline{\chi }}_{tt}}^\alpha (t,y).$

*Case (b)*: Let $\frac{\partial }{\partial y}\big [\chi (t,y)\big ]^\alpha$, $\frac{\partial }{\partial t}\big [\chi (t,y)\big ]^\alpha$ are $(i)-$differentiable and $\frac{\partial ^2}{\partial t^2}\big [\chi (t,y)\big ]^\alpha$ is $(ii)-$differentiable (OR) $\frac{\partial }{\partial y}\big [\chi (t,y)\big ]^\alpha$, $\frac{\partial }{\partial t}\big [\chi (t,y)\big ]^\alpha$ are $(ii)-$differentiable and $\frac{\partial ^2}{\partial t^2}\big [\chi (t,y)\big ]^\alpha$ is $(i)-$differentiable then using Theorem (3.1), in Eq. (5.2), one can get

5.7 $$\begin{aligned} \left\{ \begin{aligned} {\mathcal {N}}\left\{\frac{\partial }{\partial y}{{\underline{\chi }}}^\alpha (t,y)\right\}= & {} \left(- \frac{1}{u}{{\overline{\chi }}_{t}}^\alpha (0,y)\right) \ominus \bigg [\left ( -\frac{s^2}{u^2} \underline{{\widehat{R}}}^\alpha (s, u, y)\right) \ominus \left( -\frac{s}{u^2}{\underline{\chi }}^\alpha (0,y)\right) \bigg ] \oplus {\mathcal {N}}\{{\underline{g}}^\alpha (t,y)\},\\ {\mathcal {N}}\left\{\frac{\partial }{\partial y}\overline{\chi }^\alpha (t,y)\right\}= & {} \left(- \frac{1}{u}{{\underline{\chi }}_{t}}^\alpha (0,y)\right) \ominus \bigg [\left (-\frac{s^2}{u^2} {\widehat{{\overline{R}}}}^\alpha (s, u, y)\right) \ominus \left(-\frac{s}{u^2}{\overline{\chi }}^\alpha (0,y)\right) \bigg ] \oplus {\mathcal {N}}\{{\overline{g}}^\alpha (t,y)\}. \end{aligned}\right. \end{aligned}$$

Using Theorem (3.2) and initial conditions in Eq.(5.7), one can get

5.8 $$\begin{aligned} \left\{ \begin{aligned} \frac{\partial }{\partial y}\underline{{\widehat{R}}}^\alpha (s, u, y)= & {} \bigg [ \frac{s^2}{u^2} \underline{{\widehat{R}}}^\alpha (s, u, y) \ominus \frac{s}{u^2}{\underline{p}}_{0}(y,\alpha ) \bigg ] \ominus \frac{1}{u}{\overline{q}}_{0}(y,\alpha ) \oplus {\mathcal {N}}\{{\underline{g}}^\alpha (t,y)\},\\ \frac{\partial }{\partial y}\overline{{\widehat{R}}}^\alpha (s, u, y)= & {} \bigg [ \frac{s^2}{u^2} {\widehat{{\overline{R}}}^\alpha }(s, u, y) \ominus \frac{s}{u^2}{\overline{p}}_{0}(y,\alpha ) \bigg ] \ominus \frac{1}{u}{\underline{q}}_{0}(y,\alpha ) \oplus {\mathcal {N}}\{{\overline{g}}^\alpha (t,y)\}. \end{aligned}\right. \end{aligned}$$

Solving Eq. (5.8) and using initial conditions, assume that solution is obtained as follows

5.9 $$\begin{aligned} \left\{ \begin{aligned} \underline{{\widehat{R}}}^\alpha (s, u, y)= & {} R_2(s,u,y,\alpha ),\\ {\widehat{{\overline{R}}}}^\alpha (s, u, y)= & {} G_2(s,u,y,\alpha ). \end{aligned}\right. \end{aligned}$$

Taking inverse natural transform of Eq. (5.9) one can find ${\underline{\chi }}^\alpha (t,y)$ and ${\overline{\chi }}^\alpha (t,y),$ as follows.

5.10 $$\begin{aligned} \left\{ \begin{aligned} {\underline{\chi }}^\alpha (t,y)= & {} {\mathcal {N}}^{-1}\{R_2(s,u,y,\alpha )\},\\ {\overline{\chi }}^\alpha (t,y)= & {} {\mathcal {N}}^{-1}\{G_2(s,u,y,\alpha )\}. \end{aligned}\right. \end{aligned}$$

The solutions of Eq. (5.10) is valid only if ${\underline{\chi }}^\alpha (t,y)\le {\overline{\chi }}^\alpha (t,y),$ ${{\underline{\chi }}_{y}}^\alpha (t,y)\le {{\overline{\chi }}_{y}}^\alpha (t,y),$ ${{\underline{\chi }}_{t}}^\alpha (t,y)\le {{\overline{\chi }}_{t}}^\alpha (t,y)$ and ${{\underline{\chi }}_{tt}}^\alpha (t,y)\le {{\overline{\chi }}_{tt}}^\alpha (t,y).$

*Case 2*: The natural transform of following two cases are in the form

5.11 $$\begin{aligned} \left\{ \begin{aligned} {\mathcal {N}}\{{{\underline{\chi }}_{y}}^\alpha (t,y)\}= & {} {\mathcal {N}}\{{{\overline{\chi }}_{tt}}^\alpha (t,y)\} \oplus {\mathcal {N}}\{{\underline{g}}^\alpha (t,y)\},\\ {\mathcal {N}}\{{{\overline{\chi }}_{y}}^\alpha (t,y)\}= & {} {\mathcal {N}}\{{{\underline{\chi }}_{tt}}^\alpha (t,y)\} \oplus {\mathcal {N}}\{{\overline{g}}^\alpha (t,y)\}. \end{aligned}\right. \end{aligned}$$

The right and left side of these equations have ${\underline{\chi }}^\alpha (t,y)$ and ${\overline{\chi }}^\alpha (t,y)$ therefore fuzzy solutions of the following two cases can be obtained by natural Adomian decomposition method.

*Case (a)*: Let $\frac{\partial }{\partial y}\big [\chi (t,y)\big ]^\alpha$ is $(i)-$differentiable and $\frac{\partial }{\partial t}\big [\chi (t,y)\big ]^\alpha$ and $\frac{\partial ^2}{\partial t^2}\big [\chi (t,y)\big ]^\alpha$ are $(ii)-$differentiable (OR) $\frac{\partial }{\partial y}\big [\chi (t,y)\big ]^\alpha$ is $(ii)-$differentiable and $\frac{\partial }{\partial t}\big [\chi (t,y)\big ]^\alpha$ and $\frac{\partial ^2}{\partial t^2}\big [\chi (t,y)\big ]^\alpha$ are $(i)-$differentiable then using Theorem (3.1) in Eq. (5.11), one can get

5.12 $$\begin{aligned} \left\{ \begin{aligned} {\mathcal {N}}\left\{\frac{\partial }{\partial y}{{\underline{\chi }}}^\alpha (t,y)\right\}= & {} -\frac{1}{u}{{\overline{\chi }}_{t}}^\alpha (0,y)\ominus \bigg [\frac{s}{u^2}{\overline{\chi }}^\alpha (0,y)\ominus \frac{s^2}{u^2} {\widehat{{\overline{R}}}^\alpha }(s, u, y)\bigg ] \oplus {\mathcal {N}}\{{\underline{g}}^\alpha (t,y)\},\\ {\mathcal {N}}\left\{\frac{\partial }{\partial y}\overline{\chi }^\alpha (t,y)\right\}= & {} -\frac{1}{u}{{\underline{\chi }}_{t}}^\alpha (0,y)\ominus \bigg [\frac{s}{u^2}{\underline{\chi }}^\alpha (0,y)\ominus \frac{s^2}{u^2} \underline{{\widehat{R}}}^\alpha (s, u, y)\bigg ] \oplus {\mathcal {N}}\{{\overline{g}}^\alpha (t,y)\}. \end{aligned}\right. \end{aligned}$$

Rearranged Eq. (5.12) for series solutions and using initial conditions, one can get

5.13 $$\begin{aligned} \left\{ \begin{aligned} \overline{{\widehat{R}}}^\alpha (s,u,y)= & {} \frac{1}{s}{\overline{p}}_{0}(y,\alpha ) \oplus \frac{u}{s^2}{\overline{q}}_{0}(y,\alpha ) \oplus \frac{u^2}{s^2}{\mathcal {N}}\{{\underline{g}}^\alpha (t,y)\} \oplus \frac{u^2}{s^2} {\mathcal {N}}\{\frac{\partial }{\partial y}{{\underline{\chi }}}^\alpha (t,y)\},\\ \underline{{\widehat{R}}}^\alpha (s,u,y)= & {} \frac{1}{s}{\underline{p}}_{0}(y,\alpha ) \oplus \frac{u}{s^2}{\underline{q}}_{0}(y,\alpha ) \oplus \frac{u^2}{s^2}{\mathcal {N}}\{{\overline{g}}^\alpha (t,y)\} \oplus \frac{u^2}{s^2} {\mathcal {N}}\{\frac{\partial }{\partial y}\overline{\chi }^\alpha (t,y)\}. \end{aligned}\right. \end{aligned}$$

Taking inverse natural transform of Eq. (5.13), one can get

5.14 $$\begin{aligned} \left\{ \begin{aligned} {\overline{\chi }}^\alpha (t,y)= & {} {\overline{p}}_{0}(y,\alpha ) \oplus t{\overline{q}}_{0}(y,\alpha ) \oplus {\mathcal {N}}^{-1} \Big [\frac{u^2}{s^2}{\mathcal {N}}\{{\underline{g}}^\alpha (t,y)\}\Big ] \oplus {\mathcal {N}}^{-1} \Big [\frac{u^2}{s^2} {\mathcal {N}}\Big \{\frac{\partial }{\partial y}{{\underline{\chi }}}^\alpha (t,y)\Big \}\Big ],\\ {\underline{\chi }}^\alpha (t,y)= & {} {\underline{p}}_{0}(y,\alpha ) \oplus t{\underline{q}}_{0}(y,\alpha ) \oplus {\mathcal {N}}^{-1} \Big [\frac{u^2}{s^2}{\mathcal {N}}\{{\overline{g}}^\alpha (t,y)\}\Big ] \oplus {\mathcal {N}}^{-1} \Big [\frac{u^2}{s^2} {\mathcal {N}}\Big \{\frac{\partial }{\partial y}\overline{\chi }^\alpha (t,y)\Big \}\Big ]. \end{aligned}\right. \end{aligned}$$

The right and left side of these equations have opposite cases ${\underline{\chi }}^\alpha (t,y)$ and ${\overline{\chi }}^\alpha (t,y)$ therefore we decompose it with Adomiam decomposition method to obtain recursive relations, with

5.15 $$\begin{aligned}{} & {} \left\{ \begin{aligned} {{\overline{\chi }}_{0}}^\alpha (t,y)= & {} {\overline{p}}_{0}(y,\alpha ) \oplus t{\overline{q}}_{0}(y,\alpha ) \oplus {\mathcal {N}}^{-1} \Big [\frac{u^2}{s^2}{\mathcal {N}}\{{\underline{g}}^\alpha (t,y)\}\Big ],\\ {{\underline{\chi }}_{0}}^\alpha (t,y)= & {} {\underline{p}}_{0}(y,\alpha ) \oplus t{\underline{q}}_{0}(y,\alpha ) \oplus {\mathcal {N}}^{-1} \Big [\frac{u^2}{s^2}{\mathcal {N}}\{{\overline{g}}^\alpha (t,y)\}\Big ]. \end{aligned}\right. \end{aligned}$$

5.16 $$\begin{aligned}{} & {} \left\{ \begin{aligned} {{\overline{\chi }}_{n+1}}^\alpha (t,y)= & {} {\mathcal {N}}^{-1} \Big [\frac{u^2}{s^2} {\mathcal {N}}\Big \{\frac{\partial }{\partial y}{{{\underline{\chi }}}_{n}}^\alpha (t,y)\Big \}\Big ],\\ {{\underline{\chi }}_{n+1}}^\alpha (t,y)= & {} {\mathcal {N}}^{-1} \Big [\frac{u^2}{s^2} {\mathcal {N}}\Big \{\frac{\partial }{\partial y}{\overline{\chi }_{n}}^\alpha (t,y)\Big \}\Big ]. \end{aligned}\right. \end{aligned}$$

Evaluating, $\big [\chi _{0}\big ]^\alpha =\big [{{\underline{\chi }}_{0}}^\alpha , {{\overline{\chi }}_{0}}^\alpha \big ],$ $\big [\chi _{1}\big ]^\alpha =\big [{{\underline{\chi }}_{1}}^\alpha , {{\overline{\chi }}_{1}}^\alpha \big ],$ $\big [\chi _{2}\big ]^\alpha =\big [{{\underline{\chi }}_{2}}^\alpha , {{\overline{\chi }}_{2}}^\alpha \big ],$... the required solution is obtained, as

$$\begin{aligned} \left\{ \begin{aligned} {\underline{\chi }}^\alpha (t,y)= & {} {\sum _{n=0}^\infty } {{\underline{\chi }}_{n}}^\alpha (t,y),\\ {\overline{\chi }}^\alpha (t,y)= & {} {\sum _{n=0}^\infty } {{\overline{\chi }}_{n}}^\alpha (t,y). \end{aligned}\right. \end{aligned}$$

The series fuzzy solutions of Eq.(5.1), can be obtained, as

$$\begin{aligned} \big [\chi (t,y)\big ]^\alpha = \big [\chi _{0}(t,y)\big ]^\alpha + \big [\chi _{1}(t,y)\big ]^\alpha + \big [\chi _{2}(t,y)\big ]^\alpha +...+ \big [\chi _{n}(t,y)\big ]^\alpha \end{aligned}$$

The solutions is valid only if ${\underline{\chi }}^\alpha (t,y)\le {\overline{\chi }}^\alpha (t,y),$ ${{\underline{\chi }}_{y}}^\alpha (t,y)\le {\overline{\chi }}_{y}(t,y),$ ${{\underline{\chi }}_{t}}^\alpha (t,y)\le {{\overline{\chi }}_{t}}^\alpha (t,y)$ and ${{\underline{\chi }}_{tt}}^\alpha (t,y)\le {{\overline{\chi }}_{tt}}^\alpha (t,y).$

*Case (b)*: Let $\frac{\partial }{\partial y}\big [\chi (t,y)\big ]^\alpha$ and $\frac{\partial ^2}{\partial t^2}\big [\chi (t,y)\big ]^\alpha ,$ are $(i)-$differentiable and $\frac{\partial }{\partial t}\big [\chi (t,y)\big ]^\alpha$ is $(ii)-$differentiable (OR) $\frac{\partial }{\partial y}\chi ^\alpha (t,y)$ and $\frac{\partial ^2}{\partial t^2}\big [\chi (t,y)\big ]^\alpha$ are $(ii)-$differentiable and $\frac{\partial }{\partial t}\big [\chi (t,y)\big ]^\alpha$ is $(i)-$differentiable then using Theorem (3.1) in Eq.(5.11), one can get

5.17 $$\begin{aligned} \left\{ \begin{aligned} {\mathcal {N}}\left\{\frac{\partial }{\partial y}{{\underline{\chi }}}^\alpha (t,y)\right\}= & {} \bigg [\left(-\frac{s}{u^2}{\overline{\chi }}^\alpha (0,y)\right)\ominus \left( -\frac{s^2}{u^2} {\widehat{{\overline{R}}}^\alpha }(s, u, y)\right)\bigg ] \ominus \frac{1}{u}{{\underline{\chi }}_{t}}^\alpha (0,y) \oplus {\mathcal {N}}\{{\underline{g}}^\alpha (t,y)\},\\ {\mathcal {N}}\left\{\frac{\partial }{\partial y}\overline{\chi }^\alpha (t,y)\right\}= & {} \bigg [\left(-\frac{s}{u^2}{\underline{\chi }}^\alpha (0,y)\right)\ominus \left(-\frac{s^2}{u^2} \underline{{\widehat{R}}}^\alpha (s, u, y)\right)\bigg ]\ominus \frac{1}{u}{{\overline{\chi }}_{t}}^\alpha (0,y) \oplus {\mathcal {N}}\{{\overline{g}}^\alpha (t,y)\}. \end{aligned}\right. \end{aligned}$$

Rearranged, Eq. (5.17) for series solutions and using initial conditions, one can get

5.18 $$\begin{aligned} \left\{ \begin{aligned} {\overline{\chi }}^\alpha (s,y)= & {} \frac{1}{s}{\overline{p}}_{0}(y,\alpha ) \oplus \frac{u}{s^2}{\underline{q}}_{0}(y,\alpha ) \oplus \frac{u^2}{s^2}{\mathcal {N}}\{{\underline{g}}^\alpha (t,y)\} \oplus \frac{u^2}{s^2} {\mathcal {N}}\left\{\frac{\partial }{\partial y}{{\underline{\chi }}}^\alpha (t,y)\right\},\\ {\underline{\chi }}^\alpha (s,y)= & {} \frac{1}{s}{\underline{p}}_{0}(y,\alpha ) \oplus \frac{u}{s^2}{\overline{q}}_{0}(y,\alpha ) \oplus \frac{u^2}{s^2}{\mathcal {N}}\{{\overline{g}}^\alpha (t,y)\} \oplus \frac{u^2}{s^2} {\mathcal {N}}\left\{\frac{\partial }{\partial y}\overline{\chi }^\alpha (t,y)\right\}. \end{aligned}\right. \end{aligned}$$

Taking inverse natural transform of Eq. (5.18), one can get

5.19 $$\begin{aligned} \left\{ \begin{aligned} {\overline{\chi }}^\alpha (t,y)= & {} {\overline{p}}_{0}(y,\alpha ) \oplus t{\underline{q}}_{0}(y,\alpha ) \oplus {\mathcal {N}}^{-1} \Big [\frac{u^2}{s^2}{\mathcal {N}}\{{\underline{g}}^\alpha (t,y)\}\Big ] \oplus {\mathcal {N}}^{-1} \Big [\frac{u^2}{s^2} {\mathcal {N}}\Big \{\frac{\partial }{\partial y}{{\underline{\chi }}}^\alpha (t,y)\Big \}\Big ],\\ {\underline{\chi }}^\alpha (t,y)= & {} {\underline{p}}_{0}(y,\alpha ) \oplus t{\overline{q}}_{0}(y,\alpha ) \oplus {\mathcal {N}}^{-1} \Big [\frac{u^2}{s^2}{\mathcal {N}}\{{\overline{g}}^\alpha (t,y)\}\Big ] \oplus {\mathcal {N}}^{-1} \Big [\frac{u^2}{s^2} {\mathcal {N}}\Big \{\frac{\partial }{\partial y}\overline{\chi }^\alpha (t,y)\Big \}\Big ]. \end{aligned}\right. \end{aligned}$$

The right and left side of these equations have opposite cases ${\underline{\chi }}^\alpha (t,y)$ and ${\overline{\chi }}^\alpha (t,y)$ therefore we decompose it with Adomiam decomposition method to obtain recursive relations, with

5.20 $$\begin{aligned} \left\{ \begin{aligned} {{\overline{\chi }}_{0}}^\alpha (t,y)= & {} {\overline{p}}_{0}(y,\alpha ) \oplus t{\underline{q}}_{0}(y,\alpha ) \oplus {\mathcal {N}}^{-1} \Big [\frac{u^2}{s^2}{\mathcal {N}}\{{\underline{g}}^\alpha (t,y)\}\Big ],\\ {{\underline{\chi }}_{0}}^\alpha (t,y)= & {} {\underline{p}}_{0}(y,\alpha ) \oplus t{\overline{q}}_{0}(y,\alpha ) \oplus {\mathcal {N}}^{-1} \Big [\frac{u^2}{s^2}{\mathcal {N}}\{{\overline{g}}^\alpha (t,y)\}\Big ]. \end{aligned}\right. \end{aligned}$$

5.21 $$\begin{aligned} \left\{ \begin{aligned} {{\underline{\chi }}_{n+1}}^\alpha (t,y)= & {} {\mathcal {N}}^{-1} \Big [\frac{u^2}{s^2} {\mathcal {N}}\Big \{\frac{\partial }{\partial y}{\overline{\chi }_{n}}^\alpha (t,y)\Big \}\Big ],\\ {{\overline{\chi }}_{n+1}}^\alpha (t,y)= & {} {\mathcal {N}}^{-1} \Big [\frac{u^2}{s^2} {\mathcal {N}}\Big \{\frac{\partial }{\partial y}{{{\underline{\chi }}}_{n}}^\alpha (t,y)\Big \}\Big ]. \end{aligned}\right. \end{aligned}$$

Evaluating, $\big [\chi _{0}\big ]^\alpha =\big [{{\underline{\chi }}_{0}}^\alpha , {{\overline{\chi }}_{0}}^\alpha \big ],$ $\big [\chi _{1}\big ]^\alpha =\big [{{\underline{\chi }}_{1}}^\alpha , {{\overline{\chi }}_{1}}^\alpha \big ],$ $\big [\chi _{2}\big ]^\alpha =\big [{{\underline{\chi }}_{2}}^\alpha , {{\overline{\chi }}_{2}}^\alpha \big ],$... the required solution is obtained, as

$$\begin{aligned} \left\{ \begin{aligned} {\underline{\chi }}^\alpha (t,y)= & {} {\sum _{n=0}^\infty } {{\underline{\chi }}_{n}}^\alpha (t,y),\\ {\overline{\chi }}^\alpha (t,y)= & {} {\sum _{n=0}^\infty } {{\overline{\chi }}_{n}}^\alpha (t,y). \end{aligned}\right. \end{aligned}$$

The series fuzzy solutions of Eq. (5.1), can be obtained, as

$$\begin{aligned} \big [\chi (t,y)\big ]^\alpha = \big [\chi _{0}(t,y)\big ]^\alpha + \big [\chi _{1}(t,y)\big ]^\alpha + \big [\chi _{2}(t,y)\big ]^\alpha +...+ \big [\chi _{n}(t,y)\big ]^\alpha \end{aligned}$$

The solutions is valid only if ${\underline{\chi }}^\alpha (t,y)\le {\overline{\chi }}^\alpha (t,y),$ ${{\underline{\chi }}_{y}}^\alpha (t,y)\le {\overline{\chi }}_{y}(t,y),$ ${{\underline{\chi }}_{t}}^\alpha (t,y)\le {{\overline{\chi }}_{t}}^\alpha (t,y)$ and ${{\underline{\chi }}_{tt}}^\alpha (t,y)\le {{\overline{\chi }}_{tt}}^\alpha (t,y).$

### Example 5.1

Here we discuss the following heat problems as an example.

5.22 $$\begin{aligned} \left\{ \begin{aligned} \big [\chi _{y}(t,y)\big ]^\alpha= & {} \big [\chi _{tt}(t,y)\big ]^\alpha \oplus y^2 + yt,\ \ \ \ \ \ \ \ \ \ \ \ \ \ \ \ \ \ \\ \chi ^\alpha (0,y)= & {} p_{0}(y,\alpha ) = y(\alpha -1, 1-\alpha ) + \frac{y^3}{3},\ \ \ \\ {\chi _{t}}^\alpha (0,y)= & {} q_{0}(y) = y(\alpha -1, 1-\alpha ) + \frac{y^2}{2}, \ \ \ \\ \chi ^\alpha (t,0)= & {} r_{0}(t,\alpha ) = (\frac{t^2}{2!} + \frac{t^3}{3!})(\alpha -1, 1-\alpha ). \end{aligned}\right. \end{aligned}$$

Where, the fuzzy function $\chi ^\alpha :[0,\infty )\times [0,\infty )\rightarrow R_F,$ is define on $y,t\ge 0.$

*Case (1)*: For the fuzzy solutions the following two cases can be solved by using appropriate integrating factor.

*Case (a)*: Let $\frac{\partial }{\partial y}\big [\chi (t,y)\big ]^\alpha$, $\frac{\partial }{\partial t}\big [\chi (t,y)\big ]^\alpha$ and $\frac{\partial ^2}{\partial t^2}\big [\chi (t,y)\big ]^\alpha$ are $(i)-$differentiable (OR) $(ii)-$differentiable then substituting, $g(t,y) = y^2 + yt,$ and initial condition in Eq. (5.4), one can get

5.23 $$\begin{aligned} \left\{ \begin{aligned} \frac{\partial }{\partial y}\underline{{\widehat{R}}}^\alpha (s, u, y)= & {} \frac{s^2}{u^2} \underline{{\widehat{R}}}^\alpha (s, u, y) \ominus \frac{s}{u^2}\{y(\alpha -1) + \frac{y^3}{3}\} \ominus \frac{1}{u}\{y(\alpha -1) + \frac{y^2}{2}\}\oplus \frac{y^2}{s} + \frac{uy}{s^2},\\ \frac{\partial }{\partial y}{\widehat{{\overline{R}}}}^\alpha (s, u, y)= & {} \frac{s^2}{u^2} {\widehat{{\overline{R}}}}^\alpha (s, u, y) \ominus \frac{s}{u^2}\{y(1-\alpha ) + \frac{y^3}{3}\} \ominus \frac{1}{u}\{y(1-\alpha ) + \frac{y^2}{2}\}\oplus \frac{y^2}{s} + \frac{uy}{s^2}. \end{aligned}\right. \end{aligned}$$

Solving Eq. (5.23), by using integrating factor $I.F = e^{-\frac{s^2}{u^2}y}$ and using initial condition

$\big [\chi (t,0)\big ]^\alpha = (\frac{t^2}{2!} + \frac{t^3}{3!}) (\alpha -1,1-\alpha ),$ one can easily obtain fuzzy solutions as follows.

5.24 $$\begin{aligned} \left\{ \begin{aligned} {\underline{\chi }}^\alpha (t,y)= & {} y(\alpha -1) + \frac{y^3}{3} + \left\{y(\alpha -1) + \frac{y^2}{2}\right\}t + (\alpha -1)\frac{t^2}{2!} + (\alpha -1)\frac{t^3}{3!},\\ {\overline{\chi }}^\alpha (t,y)= & {} y(1-\alpha ) + \frac{y^3}{3} + \left\{y(1-\alpha ) + \frac{y^2}{2}\right\}t + (1-\alpha )\frac{t^2}{2!} + (1-\alpha )\frac{t^3}{3!} \end{aligned}\right\}. \end{aligned}$$

*Case (b)*: Let $\frac{\partial }{\partial y}\big [\chi (t,y)\big ]^\alpha$, $\frac{\partial }{\partial t}\big [\chi (t,y)\big ]^\alpha$ are $(i)-$differentiable and $\frac{\partial ^2}{\partial t^2}\big [\chi (t,y)\big ]^\alpha$ is $(ii)-$differentiable (OR) $\frac{\partial }{\partial y}\big [\chi (t,y)\big ]^\alpha$, $\frac{\partial }{\partial t}\big [\chi (t,y)\big ]^\alpha$ are $(ii)-$differentiable and $\frac{\partial ^2}{\partial t^2}\big [\chi (t,y)\big ]^\alpha$ is $(i)-$differentiable then substituting $g(t,y) = y^2 + yt$ and initial conditions in Eq. (5.4), one can get

5.25 $$\begin{aligned} \left\{ \begin{aligned} \frac{\partial }{\partial y}\underline{{\widehat{R}}}^\alpha (s, u, y)= & {} \frac{s^2}{u^2} \underline{{\widehat{R}}}^\alpha (s, u, y) \ominus \frac{s}{u^2}\left\{y(\alpha -1) + \frac{y^3}{3}\right\} \ominus \frac{1}{u}\left\{y(1-\alpha ) + \frac{y^2}{2}\right\}\oplus \frac{y^2}{s} + \frac{uy}{s^2},\\ \frac{\partial }{\partial y}{\widehat{{\overline{R}}}^\alpha }(s, u, y)= & {} \frac{s^2}{u^2} {\widehat{{\overline{R}}}^\alpha }(s, u, y) \ominus \frac{s}{u^2}\left\{y(1-\alpha ) + \frac{y^3}{3}\right\} \ominus \frac{1}{u}\left\{y(\alpha -1) + \frac{y^2}{2}\right\}\oplus \frac{y^2}{s} + \frac{uy}{s^2}. \end{aligned}\right. \end{aligned}$$

Solving Eq. (5.23) by using integrating factor, $I.F = e^{-\frac{s^2}{u^2}y}$ and initial condition

$\big [\chi (t,0)\big ]^\alpha = (\frac{t^2}{2!} + \frac{t^3}{3!})(\alpha -1, 1-\alpha ),$ one can easily obtain the fuzzy solutions, as follows.

5.26 $$\begin{aligned} \left\{ \begin{aligned} {\underline{\chi }}^\alpha (t,y)= & {} y(\alpha -1) + \frac{y^3}{3} + \left\{y(1-\alpha ) + \frac{y^2}{2}\right\}t + (\alpha -1)\frac{t^2}{2!} + (1-\alpha )\frac{t^3}{3!},\\ {\overline{\chi }}^\alpha (t,y)= & {} y(1-\alpha ) + \frac{y^3}{3} + \left\{y(\alpha -1) + \frac{y^2}{2}\right\}t + (1-\alpha )\frac{t^2}{2!} + (\alpha -1)\frac{t^3}{3!} \end{aligned}\right\}. \end{aligned}$$

*Case (2)*: The following two cases are difficult to solved by integrating factor therefore, we apply Adomiam decomposition method.

*Case(a)*: Let $\frac{\partial }{\partial y}\big [\chi (t,y)\big ]^\alpha$ is $(i)-$differentiable and $\frac{\partial }{\partial t}\big [\chi (t,y)\big ]^\alpha$ and $\frac{\partial ^2}{\partial t^2}\big [\chi (t,y)\big ]^\alpha$ are $(ii)-$differentiable (OR) $\frac{\partial }{\partial y}\big [\chi (t,y)\big ]^\alpha$ is $(ii)-$differentiable and $\frac{\partial }{\partial t}\big [\chi (t,y)\big ]^\alpha$ and $\frac{\partial ^2}{\partial t^2}\big [\chi (t,y)\big ]^\alpha$ are $(i)-$differentiable then using initial conditions in Eq. (5.15) in Eq. (5.16) one can get easily the following series

5.27 $$\begin{aligned} \left\{ \begin{aligned} {{\overline{\chi }}}^\alpha (t,y)= & {} y(1-\alpha ) + \frac{y^3}{3} + \left\{y(1-\alpha ) + \frac{y^2}{2}\right\}t - \frac{t^2 y^2}{2!} - \frac{t^3 y}{3!} \oplus \frac{u^2}{s^2} {\mathcal {N}}\left\{\frac{\partial }{\partial y}{{\underline{\chi }}}^\alpha (t,y)\right\},\\ {{\underline{\chi }}_{0}}^\alpha (t,y)= & {} y(\alpha -1) + \frac{y^3}{3} + \left\{y(\alpha -1) + \frac{y^2}{2}\right\}t - \frac{t^2 y^2}{2!} - \frac{t^3 y}{3!} \oplus \frac{u^2}{s^2} {\mathcal {N}}\left\{\frac{\partial }{\partial y}\overline{\chi }^\alpha (t,y)\right\}. \end{aligned}\right. \end{aligned}$$

The right and left side of these equations have opposite cases ${\underline{\chi }}^\alpha (t,y)$ and ${\overline{\chi }}^\alpha (t,y)$ therefore we decompose it with Adomiam decomposition method to obtain recursive relations, with

$$\begin{aligned}{} & {} \left\{ \begin{aligned} {{\overline{\chi }}_{0}}^\alpha (t,y)= & {} y(1-\alpha ) + \frac{y^3}{3} + \{y(1-\alpha ) + \frac{y^2}{2}\}t - \frac{t^2 y^2}{2!} - \frac{t^3 y}{3!},\\ {{\underline{\chi }}_{0}}^\alpha (t,y)= & {} y(\alpha -1) + \frac{y^3}{3} + \{y(\alpha -1) + \frac{y^2}{2}\}t - \frac{t^2 y^2}{2!} - \frac{t^3 y}{3!}. \end{aligned}\right. \\{} & {} \left\{ \begin{aligned} {{\overline{\chi }}_{1}}^\alpha (t,y)= & {} {\mathcal {N}}^{-1} \Big [\frac{u^2}{s^2} {\mathcal {N}}\Big \{\frac{\partial }{\partial y}\Big ( y(\alpha -1) + \frac{y^3}{3} + \{y(\alpha -1) + \frac{y^2}{2}\}t - \frac{t^2 y^2}{2!} - \frac{t^3 y}{3!}\Big )\Big \}\Big ],\\ {{\underline{\chi }}_{1}}^\alpha (t,y)= & {} {\mathcal {N}}^{-1} \Big [\frac{u^2}{s^2} {\mathcal {N}}\Big \{\frac{\partial }{\partial y}\Big (y(1-\alpha ) + \frac{y^3}{3} + \{y(1-\alpha ) + \frac{y^2}{2}\}t - \frac{t^2 y^2}{2!} - \frac{t^3 y}{3!}\Big )\Big \}\Big ]. \end{aligned}\right. \\{} & {} \left\{ \begin{aligned} {{\overline{\chi }}_{1}}^\alpha (t,y)= & {} \frac{t^2}{2!}(\alpha -1 + y^2) + \frac{t^3}{3!}(\alpha -1 + y) - \frac{2t^4 y}{2!} - \frac{t^5}{5!},\\ {{\underline{\chi }}_{1}}^\alpha (t,y)= & {} \frac{t^2}{2!}(1-\alpha + y^2) + \frac{t^3}{3!}(1-\alpha + y) - \frac{2t^4 y}{2!} - \frac{t^5}{5!}. \end{aligned}\right. \\{} & {} \left\{ \begin{aligned} {{\overline{\chi }}_{2}}^\alpha (t,y)= & {} {\mathcal {N}}^{-1} \Big [\frac{u^2}{s^2} {\mathcal {N}}\Big \{\frac{\partial }{\partial y}\Big (\frac{t^2}{2!}(1-\alpha + y^2) + \frac{t^3}{3!}(1-\alpha + y) - \frac{2t^4 y}{2!} - \frac{t^5}{5!}\Big )\Big \}\Big ] = \frac{2yt^4}{4!} + \frac{t^5}{5!} - \frac{2t^6}{6},\\ {{\underline{\chi }}_{2}}^\alpha (t,y)= & {} {\mathcal {N}}^{-1} \Big [\frac{u^2}{s^2} {\mathcal {N}}\Big \{\frac{\partial }{\partial y}\Big (\frac{t^2}{2!}(\alpha -1 + y^2) + \frac{t^3}{3!}(\alpha -1 + y) - \frac{2t^4 y}{2!} - \frac{t^5}{5!}\Big )\Big \}\Big ] = \frac{2yt^4}{4!} + \frac{t^5}{5!} - \frac{2t^6}{6}. \end{aligned}\right. \\{} & {} \left\{ \begin{aligned} {{\overline{\chi }}_{3}}^\alpha (t,y)= & {} {\mathcal {N}}^{-1} \Big [\frac{u^2}{s^2} {\mathcal {N}}\Big \{\frac{\partial }{\partial y}\Big (\frac{2yt^4}{4!} + \frac{t^5}{5!} - \frac{2t^6}{6}\Big )\Big \}\Big ] = \frac{2t^6}{6},\\ {{\underline{\chi }}_{3}}^\alpha (t,y)= & {} {\mathcal {N}}^{-1} \Big [\frac{u^2}{s^2} {\mathcal {N}}\Big \{\frac{\partial }{\partial y}\Big (\frac{2yt^4}{4!} + \frac{t^5}{5!} - \frac{2t^6}{6}\Big )\Big \}\Big ] = \frac{2t^6}{6}. \end{aligned}\right. \end{aligned}$$

The fuzzy solutions of Eq.(5.22), can be obtained as

$$\begin{aligned}{} & {} \big [\chi (t,y)\big ]^\alpha = \big [\chi _{0}(t,y)\big ]^\alpha + \big [\chi _{1}(t,y)\big ]^\alpha + \big [\chi _{2}(t,y)\big ]^\alpha + \big [\chi _{3}(t,y)\big ]^\alpha .\\{} & {} \left\{ \begin{aligned} {\underline{\chi }}^\alpha (t,y)= & {} y(\alpha -1) + \frac{y^3}{3} + \{y(\alpha -1) + \frac{y^2}{2}\}t + (1-\alpha )\frac{t^2}{2!} + (1-\alpha )\frac{t^3}{3!},\\ {\overline{\chi }}^\alpha (t,y)= & {} y(1-\alpha ) + \frac{y^3}{3} + \{y(1-\alpha ) + \frac{y^2}{2}\}t + (\alpha -1)\frac{t^2}{2!} + (\alpha -1)\frac{t^3}{3!}\}. \end{aligned}\right. \end{aligned}$$

*Case (b)*: Let $\frac{\partial }{\partial y}\big [\chi (t,y)\big ]^\alpha$ and $\frac{\partial ^2}{\partial t^2}\big [\chi (t,y)\big ]^\alpha$ are $(i)-$differentiable and $\frac{\partial }{\partial t}\big [\chi (t,y)\big ]^\alpha$ is $(ii)-$differentiable (OR) $\frac{\partial }{\partial y}\big [\chi (t,y)\big ]^\alpha$ and $\frac{\partial ^2}{\partial t^2}\big [\chi (t,y)\big ]^\alpha$ are $(ii)-$differentiable and $\frac{\partial }{\partial t}\big [\chi (t,y)\big ]^\alpha$ is $(i)-$differentiable then using initial conditions in Eq. (5.20) and Eq. (5.21), one can get easily the following fuzzy solutions by the similar procedure.

$$\begin{aligned} \left\{ \begin{aligned} {\underline{\chi }}^\alpha (t,y)= & {} y(1-\alpha ) + \frac{y^3}{3} + \{y(\alpha -1) + \frac{y^2}{2}\}t + (1-\alpha )\frac{t^2}{2!} + (\alpha -1)\frac{t^3}{3!},\\ {\overline{\chi }}^\alpha (t,y)= & {} y(\alpha -1) + \frac{y^3}{3} + \{y(1-\alpha ) + \frac{y^2}{2}\}t + (\alpha -1)\frac{t^2}{2!} + (1-\alpha )\frac{t^3}{3!}\}. \end{aligned}\right. \end{aligned}$$

## Conclusion and future direction

Fuzzy functions may have two possible generalized differentiability. FPDEs may have the possibility that the function is (i)-differentiable with respect to one variable and (ii)-differentiable with respect to the second variable. Therefore, even a FPDEs is linear may have complexities to solve it with integral transforms. By addressing the complexities associated with integral transforms used for the solutions of FPDEs, specifically, we apply the natural Adomian decomposition method to solve fuzzy advection-diffusion equations and fuzzy heat equations. This procedure is effective in dealing with first and second order linear and non-linear fuzzy partial differential equations such as advection equations, convection-diffusion equations, equations of Oscillators, nonlinear coupled partial differential equations etc.

## Data Availability

All data generated or analysed during this study are included in this published article [and its supplementary information files].
